# STAT6 Transcription Factor Is a Facilitator of the Nuclear Receptor PPARγ-Regulated Gene Expression in Macrophages and Dendritic Cells

**DOI:** 10.1016/j.immuni.2010.11.009

**Published:** 2010-11-24

**Authors:** Attila Szanto, Balint L. Balint, Zsuzsanna S. Nagy, Endre Barta, Balazs Dezso, Attila Pap, Lajos Szeles, Szilard Poliska, Melinda Oros, Ronald M. Evans, Yaacov Barak, John Schwabe, Laszlo Nagy

**Affiliations:** 1 Department of Biochemistry and Molecular Biology, University of Debrecen, Medical and Health Science Center, Research Center for Molecular Medicine, Egyetem ter 1. Debrecen, H-4010, Hungary; 2 Apoptosis and Genomics Research Group of the Hungarian Academy of Sciences, University of Debrecen, Medical and Health Science Center, Research Center for Molecular Medicine, Egyetem ter 1. Debrecen, H-4010, Hungary; 3 Department of Pathology, University of Debrecen, Medical and Health Science Center, Nagyerdei krt. 98. Debrecen, H-4028, Hungary; 4 Gene Expression Laboratory, Howard Hughes Medical Institute, The Salk Institute, La Jolla, CA 92093, USA; 5 Magee-Womens Research Institute and Department of OBGYN and Reproductive Sciences, University of Pittsburgh, 204 Craft Avenue, PA 15213, USA; 6 Department of Biochemistry, University of Leicester, Lancaster Road, Leicester, LE1 9HN, UK

## Abstract

Peroxisome proliferator-activated receptor γ (PPARγ) is a lipid-activated transcription factor regulating lipid metabolism and inflammatory response in macrophages and dendritic cells (DCs). These immune cells exposed to distinct inflammatory milieu show cell type specification as a result of altered gene expression. We demonstrate here a mechanism how inflammatory molecules modulate PPARγ signaling in distinct subsets of cells. Proinflammatory molecules inhibited whereas interleukin-4 (IL-4) stimulated PPARγ activity in macrophages and DCs. Furthermore, IL-4 signaling augmented PPARγ activity through an interaction between PPARγ and signal transducer and activators of transcription 6 (STAT6) on promoters of PPARγ target genes, including *FABP4*. Thus, STAT6 acts as a facilitating factor for PPARγ by promoting DNA binding and consequently increasing the number of regulated genes and the magnitude of responses. This interaction, underpinning cell type-specific responses, represents a unique way of controlling nuclear receptor signaling by inflammatory molecules in immune cells.

## Graphical abstract

**Graphical Abstract:**

**Figure:**
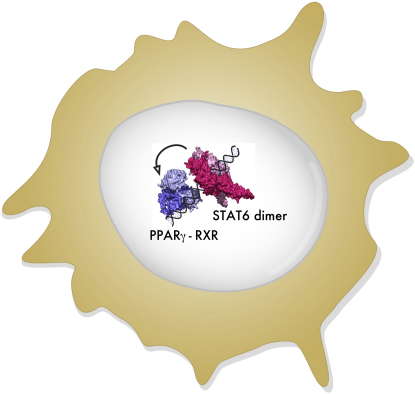

**Highlights:**

► Proinflammatory cytokines inhibit and IL-4 stimulates PPARγ activity in macrophages ► IL-4 acts as an upstream regulator of PPARγ signaling via STAT6 ► STAT6 and PPARγ bind to target gene promoters together and physically interact ► STAT6 promotes PPARγ binding and facilitates its transcriptional activity

## Introduction

PPARγ is a nuclear hormone receptor activated by oxidized fatty acids and regulating many aspects of lipid metabolism and inflammation (Itoh et al., 2008; Kliewer et al., 1997; Krey et al., 1997; Nagy et al., 1998). The major functions include regulation of adipocyte differentiation (Tontonoz et al., 1994b) and lipid metabolism in macrophages (Nagy et al., 1998; Ricote et al., 1998a; Tontonoz et al., 1998). The expression and activity of PPARγ in various cell types are strictly regulated (Kliewer et al., 1994; Szanto and Nagy, 2005; Szatmari et al., 2004; Tontonoz et al., 1994b). However, expression of the receptor and the presence of appropriate ligands are usually not sufficient to elicit optimal or maximal responses. Further transcriptional mechanisms contribute to facilitate or restrict responsiveness leading to cell type- or condition-specific gene expression pattern (Balint et al., 2005; Carroll et al., 2005; Castrillo et al., 2003; Laganière et al., 2005).

Here, we aimed to understand the impact of extracellular signals on PPARγ activity in macrophages and DCs. Both cell types represent highly specialized but heterogeneous cell populations of the immune system. Macrophages originate from bone marrow progenitors committed to the monocytic lineage (Friedman, 2002). Monocytes are recruited to sites of inflammation and turn into macrophages. The immune phenotype of macrophages depends on the cellular environment and presence of various activator molecules (Gordon, 2003). In addition to pathogen clearance they also regulate resolution of inflammatory responses. These opposing or polarized activities are initiated and maintained by immunomodulatory factors such as cytokines and microbial products and manifest in distinct activation states. Proinflammatory molecules, such as interferon-γ (IFN-γ) and tumor necrosis factor (TNF) or activators of Toll-like receptors (TLRs), result in classical activation of macrophages. In contrast, alternatively activated macrophages, which differentiate upon IL-4 stimulus (Stein et al., 1992), exhibit a different phenotype provoking tolerance or T helper 2 (Th2) immune responses (Cua and Stohlman, 1997). IL-4 induces both *Pparg* and 12/15-lipoxygenese, which synthesizes a potential ligand for PPARγ (Huang et al., 1999).

Similarly to macrophages, dendritic cells (DCs) are also capable of inducing both inflammatory and anti-inflammatory responses. DCs are sentinels of the immune system and connect innate and acquired immunity (Steinman et al., 1979). Human DCs can be modeled by monocytes exposed to granulocyte-monocyte colony stimulating factor (GM-CSF) and IL-4. This cell type has been shown to be exquisitely responsive to PPARγ activation (Gosset et al., 2001; Nencioni et al., 2002; Szatmari et al., 2004). This shared requirement of IL-4 invokes an intriguing similarity between alternatively activated macrophages and DCs. PPARγ activity has been analyzed irrespective of the inflammatory state of macrophages and DCs and prior reports focused on downstream effects of PPARγ on inflammatory reactions. Based on these, PPARγ is considered a negative regulator of macrophage activation (Jiang et al., 1998; Ricote et al., 1998b). This is believed to be mediated by the failed induction of inflammatory genes by proinflammatory transcription factors (Li et al., 2000; Pascual and Glass, 2006). However, in adipocytes, but also in DCs, PPARγ induces as well as represses hundreds of genes (Guo and Liao, 2000; Szatmari et al., 2007). These observations suggest that PPARγ responses are stringently controlled and determined by cell type and condition-specific factors. The identification of such factors could explain differences in PPARγ-evoked responses in subtypes of macrophages and DCs.

We used gene-specific and global transcriptomics approaches in mouse and human macrophage subtypes and DCs to show that proinflammatory molecules inhibited, whereas IL-4 augmented, both PPARγ expression and ligand-induced transcriptional activity. Pharmacological and genetic evidence showed that this effect was mediated by the STAT6 transcription factor, which acted as a facilitator of PPARγ-mediated transcription. In addition, we proposed a mechanism by which STAT6 interacted with PPARγ and the cooperative binding of the two factors led to increased PPARγ responsiveness. Thus, these findings provide the molecular mechanism for robust PPARγ-regulated gene expression in these cell types.

## Results

### Expression of *PPARg* Is Determined by the Activation State of Macrophages and DCs

**Figure 1:**
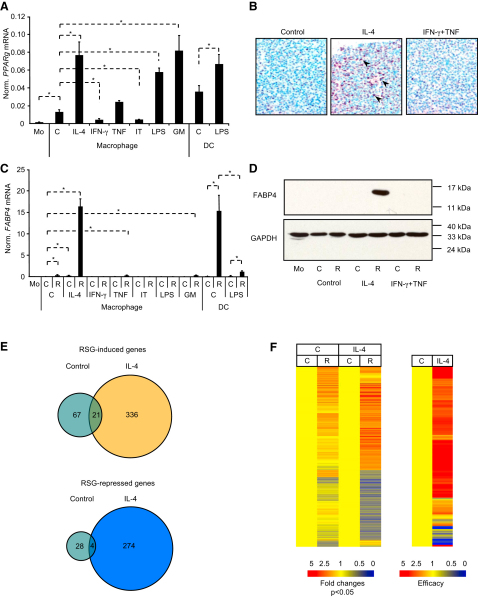
Expression and Activity of PPARγ Is Dictated by Cytokines, the Role of IL-4 (A–C) Expression of *PPARg* (A) and *FABP4* (C) was determined by real-time PCR. Human monocytes (Mo) were differentiated to macrophages for 24 hr by attachment or to DCs for 5 days with GM-CSF and IL-4. Macrophages were treated with vehicle (C), IL-4, IFN-γ, TNF, IFN-γ+TNF (IT), LPS, or GM-CSF (GM) for 24 hr. LPS was added on day 5 for 24 hr to induce DC maturation. In (C) cells were also treated with RSG (R) or vehicle (DMSO:ethanol). Means normalized to cyclophilin A ± SD. n = 3, p < 0.01 are shown. (B) PPARγ protein was determined by immunostaining with PPARγ antibody. The positive nuclear staining is indicated by purple color (arrows). Methyl-green was used as nuclear counterstain. (D) FABP4 protein was detected by immunoblot in human macrophages treated as indicated for 24 hr. Glyceraldehyde-3-phosphate dehydrogenase (GAPDH) is the loading control. (E and F) Human macrophages were cultured for 12 hr in the absence or presence of IL-4 and treated with vehicle (C) or RSG (R), gene expression was analyzed by Affymetrix Human Genome U133 2.0 Plus microarrays (n = 3). Signal intensities were normalized to the 50^th^ percentile and to the median expression of genes and to the vehicle-treated control. (E) Venn diagrams of RSG-regulated genes (>1.5-fold change, p < 0.05 parametric t test, Benjamini-Hochberg false discovery rate correction). (F) Heatmaps of 730 probe sets from (E) on the left, and the efficacy (RSG-induced fold changes in IL-4-treated versus control macrophages) is shown on the right.

To define conditions of maximal *PPARg* expression and responsiveness, human monocyte-derived macrophages were activated either with IL-4 or with the proinflammatory cytokines IFN-γ, TNF, or lipopolysaccharide (LPS). AMAC-1, CD206, CD209, and CD23 were used as markers to define alternative or CD80, CD83, CD86, and HLA-DR to define classical activation of macrophages. Immature DCs were differentiated from monocytes with GM-CSF+IL-4, and LPS was used to induce maturation. CD1a and CD209 were used as markers of DC development (data not shown) (Geijtenbeek et al., 2000; Porcelli, 1995). *PPARg* was induced during monocyte-macrophage transition (Figure 1A) and its expression was further increased by IL-4 but decreased by IFN-γ. Similarly, *PPARg* was induced upon differentiation of immature DCs and was modestly upregulated upon DC maturation with LPS (Figure 1A).

The induction of *PPARg* by IL-4 was rapid and specific and translated into increased levels of PPARγ protein in macrophages (brown nuclear staining) (Figure 1B). Neither *PPARa* nor *PPARd* showed similar expression (Figures S1A–S1C available online). In contrast, PPARγ was essentially missing from IFN-γ-stimulated classically activated cells (Figure S1B). In order to assess the in vivo expression distribution of PPARγ in macrophages, we surveyed tissues via immunohistochemistry (Figures S1E–S1Y). PPARγ-positive macrophages were identified in tissues such as Peyer's patch (Figures S1E–S1G), lamina propria of normal small intestinal villi (Figures S1H–S1J), reactive lymph node (Figures S1K–S1M), lymphoepithelial tissue of the tonsil (Figures S1N–S1P), perivascular macrophages of lymph node (Figures S1Q–S1S), and the lung (Figures S1T–S1Y), showing expression preferentially in alternatively activated macrophages as determined by DC-SIGN staining.

Next, we analyzed the ligand-induced transcriptional activity of PPARγ by treating cells with the agonist Rosiglitazone (RSG). *FABP4* (encoding FABP4, also known as aP2) (Tontonoz et al., 1994a), a well-established PPARγ target gene, was strongly induced at both mRNA and protein levels in macrophages activated with IL-4 and in GM-CSF+IL-4-induced immature DCs (Figures 1C and 1D). Modest induction of *FABP4* by RSG was observed in nonactivated control and TNF-treated macrophages and upon IL-4 treatment. Importantly, IFN-γ, IFN-γ+TNF, and/or LPS inhibited *FABP4* induction in both macrophages and mature DCs. These effects were specific for PPARγ target genes as shown by the fact that neither LXRα nor PPARα, δ activity was affected (Figure S1D and data not shown). Therefore, diverse signals could induce *PPARg* expression (e.g., LPS, GM-CSF), but only IL-4 could also augment transcriptional responsiveness as determined by target gene induction. This finding led to the hypothesis that IL-4 is a facilitator of PPARγ via a mechanism we termed IL-4-induced augmentation of PPARγ response.

### IL-4 Is an Enhancer of PPARγ Transcriptional Responses

To test the extent of IL-4 augmentation of PPARγ response, we performed microarray analysis of RSG-treated human macrophages (Figures 1E and 1F). In the absence of IL-4, RSG induced 88 and repressed 32 genes (Figure 1E). IL-4 treatment resulted in an increase in the number of both the RSG-induced (336) and -repressed (274) genes. IL-4 not only enabled PPARγ to regulate a larger set of genes (635 versus 120) but also increased the magnitude of responses on individual genes. 665 genes (out of 730 regulated by RSG in control or IL-4-treated macrophages) were more responsive to RSG in the presence of IL-4 (Figure 1F; Table S1). These data suggest that IL-4-augmented PPARγ response applies to the vast majority of PPARγ-regulated genes.

### IL-4 Induces Augmented PPARγ Response in Both Mouse Macrophages and DCs

**Figure 2:**
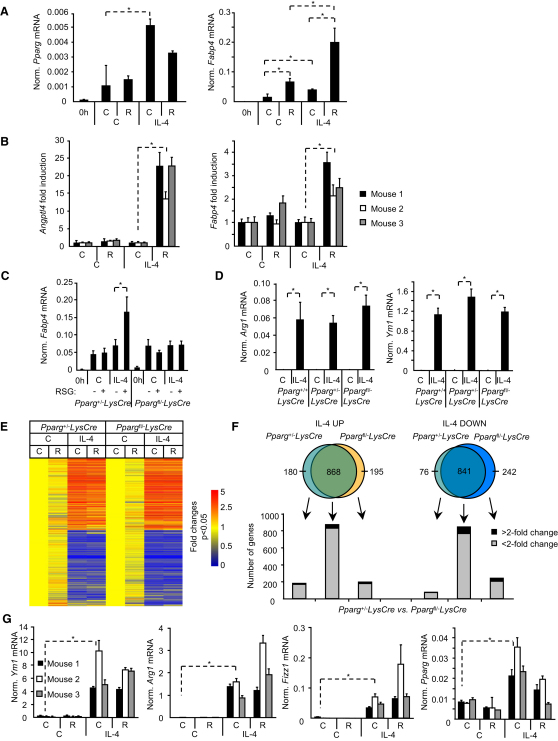
PPARγ Is Dispensable for IL-4 Signaling (A) Expression of *Pparg* (left) and *Fabp4* (right) in mouse bone marrow-derived monocytes activated as indicated was determined with real-time PCR. (B) Mouse bone marrow-derived cells (n = 3) were differentiated to macrophages for 10 days, activated as indicated, and expression of *Angptl4* (left) and *Fabp4* (right) was determined. (C and D) Expression *Fabp4* (C), *Arg1*, and *Ym1* (D) in mouse bone marrow-derived macrophages differentiated from *Pparg*^+/+^, *Pparg*^+/−^, and *Pparg*^fl/−^*LysCre* mice are shown. (E and F) Bone marrow-derived macrophages from *Pparg*^+/−^ and *Pparg*^fl/−^*LysCre* mice (n = 3) were differentiated with M-CSF or MCSF+IL-4 in the absence (C) or presence (R) of RSG for 10 days. Gene expression was analyzed by Affymetrix Mouse Genome 430 2.0 microarrays. Signal intensities were normalized to the 50^th^ percentile and to the median expression of genes and to vehicle-treated control. (E) The heatmap of the expression of IL-4-regulated genes (>2-fold, significant differences are plotted; n = 3, parametric t test, Benjamini-Hochberg false discovery rate correction, p < 0.05). (F) Venn diagrams of the IL-4-regulated genes from macrophages of *Pparg*^+/−^ and *Pparg*^fl/−^*LysCre* mice. (G) Expression of alternative activation markers, *Ym1*, *Arg1*, *Fizz1*, and that of *Pparg* from mouse bone marrow-derived macrophages. Means normalized to cyclophilin A ± SD. n = 3, p < 0.01 are shown.

IL-4 increased the expression of *Pparg* in monocytes isolated from bone marrow of C57Bl/6 mice (Figure 2A). *Fabp4* was induced by RSG and this change was augmented by IL-4 (Figure 2A). Alternative activation markers such as found in inflammatory zone 1 (*Fizz1*), mannose receptor (*Mr*), chitinase 3-like 3 (*Ym1*), and arginase 1 (*Arg1*) were induced by IL-4 (data not shown). RSG treatment barely induced the expression of PPARγ target genes, PPARγ angiopoietin-related protein (*Angptl4*), or *Fabp4* in mouse macrophages whereas the addition of IL-4 elicited responsiveness to RSG (Figure 2B). Induction of PPARγ target genes showed a similar pattern in mouse bone marrow-derived DCs (Figure S2A). These data demonstrate that human and murine macrophages and DCs behave similarly with respect to the effects of IL-4 on PPARγ expression and responsiveness.

### PPARγ Is Dispensable for IL-4 Signaling

Next, we assessed the relationship between PPARγ-IL-4 in macrophage-specific PPARγ-deficient mice carrying loxP-flanked and null alleles *Pparg*^fl/−^ (He et al., 2003) and a lysozyme (*Lys*) *Cre* transgene (Clausen et al., 1999). PCR assessment demonstrated near complete recombination (Figure S2B). This was confirmed by the complete loss of *Fabp4* induction by RSG (Figure 2C). No major differences in the induction of *Arg1* or *Ym1* markers of alternatively activated macrophages were detected in IL-4-treated bone marrow-derived macrophages from wild-type, *Pparg*^+/−^ *LysCre*, or *Pparg*^fl/−^ *LysCre* mice (Figure 2D). Similar results were obtained in peritoneal macrophages (data not shown). We performed microarray experiments to address the general contribution of PPARγ to IL-4 responses by using macrophages from *Pparg*^+/−^ and *Pparg*^fl/−^ *LysCre* mice. The vast majority of IL-4-regulated genes showed similar expression pattern. Neither the number of IL-4-regulated genes nor the magnitude of responses was affected by the absence of *Pparg* (Figures 2E and 2F; Table S2). Importantly, no difference was detected in the induction of alternative activation markers by IL-4 (Table S2). The majority of changing genes showed less than 2-fold difference between the two genotypes (*Pparg*^+/−^ and *Pparg* ^fl/−^ *LysCre*) (Figures 2E and 2F). Notably, this is also true for the nonoverlapping genes (Figure 2F). These turned out to be the lowest responders to IL-4 (1.5- to 2.5-fold) and although not significantly, the majority of them are regulated by IL-4 in both genotypes and no differentially expressed cluster could be detected in the heatmap or in the gene lists (Figure 2E; Table S2).

Very little influence of PPARγ was found when we compared the effects of RSG on IL-4-regulated genes and analyzed the coregulated ones (Figure 2E; Figure S2C). These data indicate only a modest contribution of PPARγ to IL-4 signaling and are inconsistent with PPARγ being required per se for initiation of alternative activation. The induction of markers of alternative macrophage activation *Ym1*, *Arg1*, or *Fizz1* by IL-4 were not affected by RSG treatment (Figure 2G). This was in agreement with the global gene expression analysis. We obtained similar results in Th2 cell-type response-prone BALB/c mice and in peritoneal macrophages (data not shown). Taken together, these data suggest that PPARγ is largely dispensable for IL-4-regulated gene expression in macrophages.

### STAT6 Is Required for PPARγ-Induced Gene Expression

**Figure 3:**
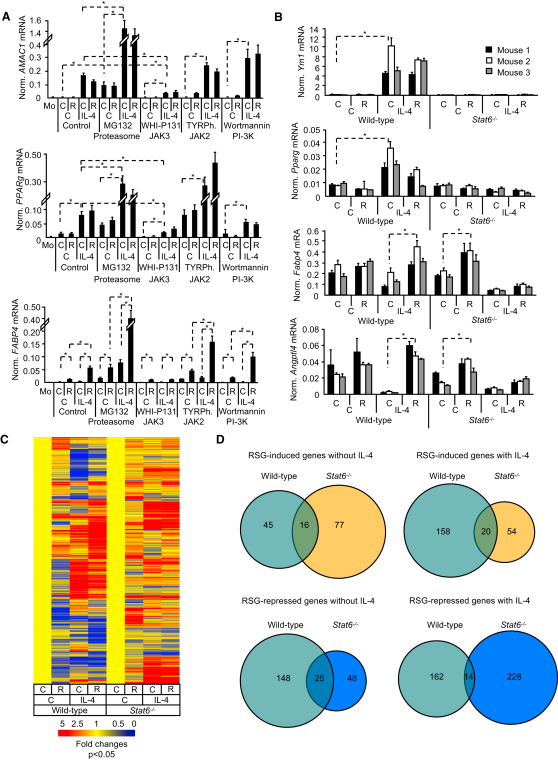
IL-4 Acts through STAT6 to Induce Augmented PPARγ Response (A) Human nonactivated or alternatively activated (IL-4) macrophages were exposed to vehicle (C), proteasome inhibitor MG132, JAK3 inhibitor WHI-P131, JAK2 inhibitor TyrPhostin AGN490 (TyrPh.), or PI-3K inhibitor wortmannin for 6 hr. Simultaneously, cells were treated with vehicle (C) or RSG. Expression of *AMAC1*, *PPARg*, and *FABP4* are shown. (B) Expression of *Ym1*, *Pparg*, *Fabp4*, and *Angptl4* was analyzed in bone marrow-derived macrophages isolated from wild-type C56Bl/6 and *Stat6*^−/−^ mice and treated for 10 days as indicated. (C and D) Mouse bone marrow-derived macrophages from wild-type C57Bl/6 and *Stat6*^−/−^ mice were cultured in the presence of vehicle (C), RSG (R), IL-4, or IL-4+RSG. Gene expression was analyzed by Affymetrix Mouse Genome 430 2.0 microarrays. Signal intensities were normalized to the 50^th^ percentile and to the median expression of the gene throughout the experiment and finally to its specific vehicle-treated control. (C) The heatmap of RSG-induced and repressed genes in the control or IL-4-treated wild-type macrophages. (D) Venn diagrams of RSG-regulated genes. Means normalized to cyclophilin A ± SD. n = 3, p < 0.01 are shown.

To identify which downstream effectors of IL-4 impact PPARγ expression and/or activity, we used pharmacological inhibitors that distinguish between STAT6 and insulin receptor substrate-2 (IRS-2) pathways. Janus Tyrosine Kinase 3 (JAK3) inhibitor WHI-P131, but not JAK2 inhibitor TYRPhostin (or AG490) or phosphatidilinositol-3 kinase (PI3K) inhibitor wortmannin, inhibited induction of an IL-4-regulated gene, *AMAC1*, and of *PPARg*, indicating that these effects of IL-4 are mediated by JAK3 (Figure 3A). Similarly, JAK3 but not the JAK2 and PI3K inhibitors inhibited IL-4-induced augmentation of *FABP4* induction by RSG (Figure 3A). These results are consistent with the report that JAK3 is the major JAK isoform in myeloid cells (Witthuhn et al., 1994) and implicated its substrate STAT6 as the downstream IL-4 effector.

Next, we derived bone marrow-derived macrophages from wild-type and STAT6-deficient mice. Induction of the alternative activation marker *Ym1* and *Pparg* by IL-4 shows STAT6 dependence (Figure 3B). IL-4-augmented induction of PPARγ target genes *Fabp4* and *Angptl4* by RSG was detected only in wild-type but not in STAT6-deficient macrophages (Figure 3B). To test whether STAT6 is required for PPARγ-mediated gene expression on a global scale, we performed microarray analyses and identified RSG-regulated genes in the absence or presence of IL-4 in wild-type and STAT6-deficient mice (Figures 3C and 3D). The absence of STAT6 had a major impact on PPARγ-regulated gene expression. Substantially more genes were induced in the presence of IL-4 (Figure 3D, upper panels 45 versus 158) and the fold inductions were larger (Figure 3C). Moreover, the vast majority (82%) (45+158 genes) of the 225 RSG-induced genes were regulated by PPARγ in a STAT6-dependent manner and 99 out of the 225 RSG-induced genes showed higher expression upon IL-4 treatment (Table S3). 125 RSG-induced genes were repressed by IL-4, indicating that the expression pattern of *Angptl4* (i.e., repression by STAT6 and activation by PPARγ; Figure 3B) is not a gene-specific phenomenon but rather characteristic of a set of genes. Interestingly, IL-4 did not increase the number of genes repressed by RSG: 173 in the control versus 176 in the IL-4-treated cells. However, out of the 318 genes RSG repressed, 70% showed STAT6 dependence (Figure 3C). *Stat6* deletion decreased the number of RSG-silenced genes in the absence of IL-4 (Figure 3D, lower panels 148 versus 48), whereas they were increased in the presence of IL-4 (162 versus 228), indicating the existence of further STAT6-independent silencing mechanisms. Taken together, these data suggest that IL-4 augments PPARγ activity via STAT6. Furthermore, STAT6 is required for induction of the majority of PPARγ target genes (82%). These data are consistent with a general, facilitating role for STAT6.

### STAT6 Augments PPARγ Activity on Target Gene Promoters

**Figure 4:**
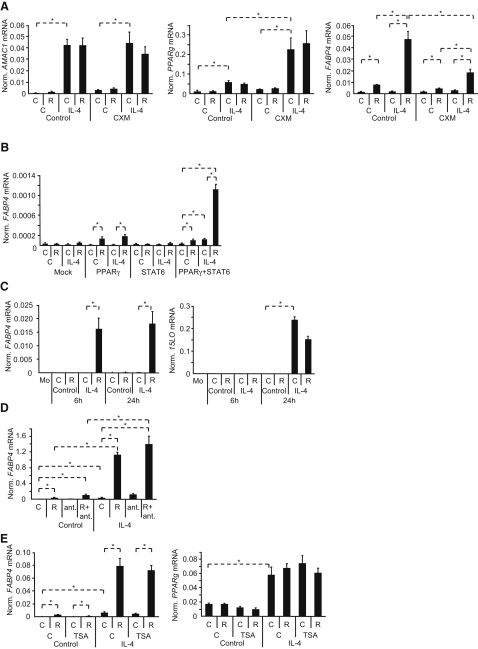
STAT6 Acts on PPARγ Response at the Transcriptional Level (A) Human macrophages were treated with vehicle or CXM, activated with vehicle (C) or IL-4 for 6 hr, and simultaneously vehicle (C) or RSG (R) was added. Expression of *AMAC1*, *PPARg*, and *FABP4* were measured with real-time PCR. (B) 293T cells were transfected with mock, *PPARg*, *STAT6*, or *PPARg*+*STAT6* expression vectors and treated as indicated for 24 hr and expression of *FABP4* was analyzed by real-time PCR. (C) Human macrophages were treated as indicated. Expression of *FABP4* and 15-lipoxygenase were measured by real-time PCR. (D) Human macrophages were treated with vehicle (C) or IL-4 and simultaneously with vehicle (C), RSG (R) or LG1208 RXR antagonist (ant.) for 24 hr. Expression of *FABP4* was analyzed by real-time PCR. (E) Human macrophages were treated with vehicle (C) or 100 nM TSA, activated with vehicle (C) or IL-4 for 12 hr, and simultaneously vehicle (C) or RSG (R) was also added. Expression of *FABP4* (left) and *PPARg* (right) were measured by real-time PCR. Means normalized to 36B4 (A, B, C) cyclophilin A (D, E) ± SD. n = 3, p < 0.01 are shown.

We took several possible mechanisms of STAT6-mediated PPARγ facilitation into consideration. An obvious one is to see whether new protein synthesis was required for the enhancing effect or if it is purely transcriptional. IL-4-dependent induction of a STAT6-regulated gene, *AMAC1*, and that of *PPARg* was not affected by cycloheximide (CXM), suggesting a direct transcriptional event (Figure 4A). As expected, RSG could activate PPARγ independently of new protein synthesis as reflected in *FABP4* induction (Figure 4A). In the presence of CXM, IL-4 could still enhance ligand-induced *FABP4* expression, suggesting that STAT6-augmented PPARγ response did not require new protein synthesis. However, *FABP4* induction slightly decreased, indicating the contribution of new protein synthesis, most probably PPARγ protein production upon IL-4 treatment. In order to evaluate the contribution of the increased expression of the receptor, we transiently transfected cells to overexpress PPARγ. PPARγ target gene expression was enhanced by STAT6 when cotransfected and activated by IL-4, which should be independent of the induction of PPARγ (Figure 4B and data not shown).

To prove that IL-4 directly influences PPARγ, we excluded some obvious indirect mechanisms. IL-4 was shown to increase the production of a PPARγ activator, 15d-PGJ_2_, via inducing 12/15-lipoxygenase (Huang et al., 1999). IL-4 augmented PPARγ response well before the induction of 15-lipoxygenase in human macrophages, suggesting that STAT6 is unlikely to act via ligand generation (Figure 4C). We also excluded that STAT6 would generate an activator for the retinoid X receptor, the permissive dimerization partner for PPARγ, by using an RXR antagonist (Figure 4D). We also tested trichostatin A (TSA), a histone deacetylase inhibitor, but no difference could be observed in the nonactivated cells, suggesting that IL-4 does not act via suspension of histone deacetylation (Figure 4E). Further possible mechanism could be that STAT6 induces degradation of a repressor for *PPARg* or synthesis of an activator. By using a proteasome inhibitor, MG132, and translation inhibitor CHX (Figures 3A–4A), we excluded these possibilities as well. Interestingly, MG132 not only did not inhibit the enhancement but it further increased it, suggesting the presence of an activating factor that is degraded upon activation of PPARγ-STAT6. Such factor could be either the transcription factor itself or its coactivators. We cannot exclude the possibility that other inhibitory factors exist, which are activated through the proteasomal pathway. These experiments left us with the likely possibility that STAT6 acts on the promoter of PPARγ target genes.

### STAT6 Facilitates PPARγ Signaling at the Transcriptional Level

**Figure 5:**
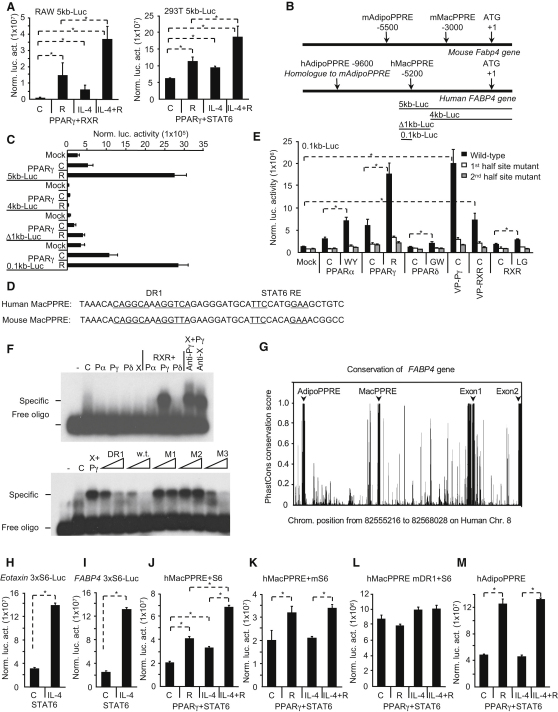
Identification of a Composite PPARγ-STAT6 Response Element in the *FABP4* Promoter (A) A reporter construct containing 5 kb fragment of human *FABP4* promoter was cotransfected into RAW264.7 and 293T cells with the indicated expression vectors, and normalized luciferase activity was determined 24 hr after cytokine or ligand treatment (control [C] or RSG [R]). (B) Schematic structure of the mouse and human *FABP4* with the localization of Adipo and MacPPREs. (C) Deletion mutants of the 5 kb promoter of the human *FABP4* were cotransfected into 293T cells with mock or PPARγ expression vectors. Normalized luciferase activity was determined 24 hr after ligand treatment (vehicle [C] or RSG [R]). (D) Sequences of human and mouse MacPPREs of the *Fabp4* gene. (E) The human MacPPRE was mutated at the 1^st^ or 2^nd^ half site and tested in transfection assays. Reporter constructs were cotransfected into 293T cells with the indicated receptor, *VP16-PPARg* (VP-Pγ), *VP16-RXR* (VP-RXR) expression vectors. Normalized luciferase activity was determined 24 hr after ligand treatment (vehicle [C], WY14643 [WY], RSG [R], GW501516 [GW], or LG268 [LG]). (F) DNA binding of PPARα (Pα), γ (Pγ), δ (Pδ), and RXR (X) to the human MacPPRE was analyzed by EMSA. Cold competitors of consensus DR1 (DR1), wild-type MacPPRE, or its mutants (1^st^ half site-M1, 2^nd^ half site-M2, downstream sequence outside the DR1-M3) were used. (G) Conservation of human *FABP4* gene was analyzed by PhastCons conservation scores for placental mammals. The plotted region corresponds to the hg18 chromosome 8 genomic position from 82555216 to 82568028 on the negative strand. The y axis shows the PhastCons conservation scores (in the range 0–1) for each position. (H and I) Reporter constructs with three copies of STAT6 response elements of human *CCL11* (H) or *FABP4* (I) gene were transfected into 293T cells with *STAT6* and normalized luciferase activity was determined 24 hr after IL-4 (100 ng/ml) exposure. (J–M) Composite response elements of *FABP4* gene containing both PPRE and STAT6 binding sites were transfected into 293T cells along with *PPARg* and *STAT6* expression vectors. Human MacPPRE (J), STAT6 (K), or PPARγ (L) binding site mutant of MacPPRE and human AdipoPPRE (M) were tested. Normalized reporter activities as means ± SD, n = 3, p < 0.01 are shown.

We chose the prototypic target gene, *FABP4* (Tontonoz et al., 1994a), to study PPARγ response at the promoter level. A 5 kb fragment of the human promoter responded to PPARγ activators and also to IL-4 and this latter could augment the effect of RSG in a reporter assay in two different cell lines, RAW264.7 and 293T (Figure 5A). This was surprising, because this fragment did not contain the human ortholog of the originally identified PPARγ response element, which we term here AdipoPPRE (Figure 5B; Tontonoz et al., 1994a). By using deletions and mutations, we identified a response element for PPARγ:RXR (Figures 5C and 5D and data not shown). We termed this element MacPPRE referring to macrophages (Figure 5D). Electrophoretic mobility shift assays (EMSA) and reporter assays were carried out to show preferential activation and binding of PPARγ to the enhancers (Figures 5E and 5F). This specificity disappeared when we mutated the PPARγ binding site to the consensus AGGTCA (Figure S3A). The human ortholog hAdipoPPRE (Tontonoz et al., 1994a) exhibited similar enhancer activities as the hMacPPRE (Figures S3B and S3C). Interestingly, the two most conserved regions in the entire promoter region in mammals are the Adipo and MacPPREs along with the core promoter indicating their functional importance (Figure 5G; Figure S3C). Unexpectedly, we found a consensus conserved STAT6 binding site downstream to MacPPRE (Figure 5D; Figure S3C), which was not present in the proximity of AdipoPPRE. This STAT6 response element was functional and as efficient (Figure 5H) as a known STAT6 enhancer from the *CCL11* gene (Figure 5I; Matsukura et al., 1999). A short promoter fragment that contained the composite element (MacPPRE and the STAT6 element) (Figure 5J) behaved similarly as the original 5 kb fragment (Figure 5A), indicating that this fragment is responsible for the STAT6-augmented PPARγ response. Mutation of the STAT6 binding site resulted in the loss of responsiveness to IL-4 (Figure 5K) without affecting induction by RSG. Mutation of DR1 abolished RSG-induced activation and also almost completely eliminated the effects of IL-4 (Figure 5L). The hAdipoPPRE did not show IL-4 responsiveness (Figure 5M). When isolated DR1s (consensus or MacPPRE from FABP4) were tested, STAT6 was ineffective in enhancing transcriptional activity (Figures S5E and S5F). Similarly, activity of a Gal-fusion PPARγ could not be augmented by STAT6 (Figure S5G). Thus these results indicate the requirement for the STAT6 binding site in the composite element to augment PPARγ response.

### In Vivo Binding of STAT6 to PPREs

**Figure 6:**
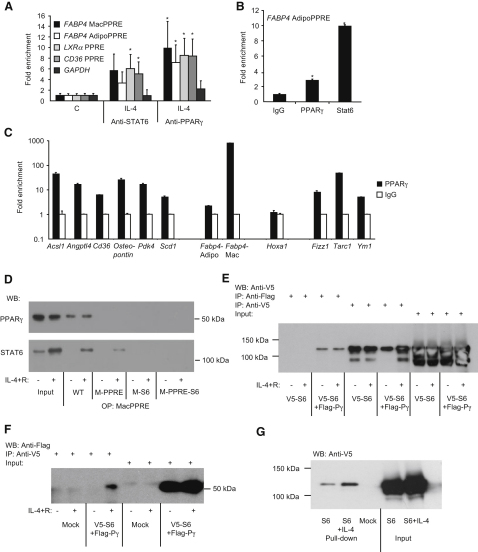
STAT6 Colocalizes and Interacts with PPARγ In Vivo and In Vitro (A) 293T cells were transfected with *PPARg* and *STAT6* expression vectors and ChIP was performed with anti-STAT6 and anti-PPARγ. IL-4-induced enrichment of *FABP4* MacPPRE, AdipoPPRE, *LXRa*-PPRE, and *CD36*-PPRE elements were analyzed by real-time PCR. *GAPDH* promoter was used as negative control. Enrichment over IgG and over control samples is presented. (B) PPARγ and STAT6 ChIP was performed on bone marrow-derived macrophages from wild-type and *Stat6*^−/−^ mice. Enrichment of *Fabp4* PPRE over the IgG and over the wild-type samples is presented. (C) PPARγ ChIP was performed on bone marrow-derived macrophages from wild-type and *Stat6*^−/−^ mice. Enrichment of PPREs (*Acsl1*, *Angptl4*, *Cd36*, osteopontin, pyruvate dehydrogenase kinase-4 [*Pdk4*], stearoyl-Coenzyme A desaturase-1 [*Scd1*], *Fabp4* Adipo-, and MacPPRE), STAT6 response elements of *Fizz1*, *Tarc1*, and *Ym1*, and the negative control *Hoxa1* over the IgG and over the wild-type samples is presented. (D) hMacPPRE coprecipitates endogenous STAT6 and PPARγ from THP-1 cells. Biotin-labeled oligonucleotides corresponding to wild-type (WT), PPRE (M-PPRE), STAT6 site (M-S6), or double mutant (M-PPRE-S6) MacPPRE were incubated with equal amounts of nuclear extracts harvested from vehicle (−) or IL-4+RSG (IL-4+R)-treated (+) cells. Pulled down proteins were immunoblotted with the indicated antibodies. Input represents 1.67% of oligoprecipitated material. (E and F) 293T cells were transfected with mock, *V5-STAT6* (V5-S6), or *V5-STAT6*+*Flag-PPARg* (V5-S6+Flag-Pγ) as indicated. Coimmunoprecipitation from whole cell lysates was performed with V5 or Flag antibodies and presence of V5-STAT6 (E) and Flag-PPARγ (F) were analyzed by immunoblotting. (G) PPARγ was expressed in bacteria and purified with streptavidine-resin, and STAT6 was pulled down from whole cell lysates of mock or V5-STAT6-transfected 293T cells. Proteins were analyzed by immunoblotting with V5 antibody. Means normalized to input ± SD. n = 3, p < 0.05 are shown.

These in vitro and transfection-based analyses established the presence of two functional PPREs in the FABP4 promoter, one of which (MacPPRE) is a complex element conferring IL-4-augmented PPARγ responsiveness. Next, we extended our studies to observe in vivo occupancy of this element by endogenous PPARγ and STAT6, and also to see how widespread the interaction of two factors is on known PPREs and STAT6 binding sites. Chromatin immunoprecipitation (ChIP) showed IL-4-induced binding of PPARγ and STAT6 to PPREs in 293T cells transfected with PPARγ and STAT6 (Figure 6A). In addition, both PPARγ and STAT6 were enriched on AdipoPPRE in wild-type macrophages when compared to *Stat6*^−/−^ cells (Figure 6B). Furthermore, with quantitative ChIP analysis on several known PPREs and STAT6 binding sites, we could detect PPARγ binding to all the tested elements except the negative control *Hoxa1*, and importantly this binding was markedly enriched in wild-type animals when compared to *Stat6*^−/−^ macrophages (Figure 6C), indicating that the two transcription factors are likely to be in the same DNA binding complex in vivo. Importantly, MacPPRE was more sensitive to the presence of STAT6 than AdipoPPRE. Thus, STAT6 seems to be enriched on several PPREs in vivo, suggesting a functional interaction between STAT6 and PPARγ.

### STAT6 Facilitates PPARγ's DNA Binding and Interacts with the Receptor

The binding of STAT6 to PPREs in PPARγ target genes' promoter and the close proximity of STAT6 and PPARγ binding sites in the human *FABP4* promoter raised the possibility of physical interaction between the two transcription factors. We first performed oligoprecipitation experiments in a monocytic leukemia cell line, THP-1, and both endogenously expressed PPARγ and STAT6 could be pulled down with wild-type MacPPRE (Figure 6D). STAT6 binding was detected only after IL-4 administration. Importantly, mutations in either PPRE or STAT6 binding sites diminished PPARγ binding, whereas STAT6 binding was eliminated only if STAT6 site was mutated. Finally, the interaction of PPARγ and STAT6 could be also detected by coimmunoprecipitation with tagged, expressed proteins (Figures 6E and 6F). STAT6 could be pulled down with PPARγ (Figure 6E) and vice versa (Figure 6F). STAT6 could also be pulled down with purified PPARγ protein (Figure 6G). Although there is a bit of inconsistency regarding the ligand dependency of PPARγ and STAT6 interaction, three out of four experiments (Figures 6D, 6F, and 6G) support that their interaction is ligand dependent. These data suggest that PPARγ and STAT6 bind the response element in *FABP4* promoter in vivo. Additionally, STAT6, by interacting with PPARγ, facilitates and is required for efficient endogenous PPARγ binding.

## Discussion

A key issue in immunology is to generate specific cell types often with opposing activities. It is of importance to understand the molecular details of the transcriptional mechanisms leading to the development of such subtypes. We found a diverse pattern of PPARγ expression and activity among the various macrophage and DC subtypes, equipping the cells with differential ability to respond to certain lipid signals. Although several agents could induce the transcription of *PPARg* (IL-4, LPS, transforming growth factor beta), only IL-4 was capable of augmenting its activity. This enhancement was reflected in the number of genes and the magnitude of responses. These data are in agreement with previous reports documenting very few positively regulated genes and a lower level of responses under proinflammatory conditions induced by LPS or IFN-γ (Welch et al., 2003) and suggested the existance of both positive and negative interactions between PPARγ and cytokine signaling. PPARγ has been described as a negative regulator of macrophage activation by transrepression (Pascual et al., 2005). However, this mechanism is unlikely to play a role here. PPARγ was also reported to be required for maturation of alternatively activated macrophages and disruption of the gene impaired alternative macrophage-linked functions in mice (Odegaard et al., 2007). Formally, these data suggested that PPARγ acts upstream of IL-4 signaling. We note that Odegaard et al. used BALB/c mice with *Mx-Cre* and rather focused on secondary effects of IL-4 signaling. Their data might reflect strain-specific differences and/or involvement of additional complex feedback mechanisms. However, our results are in agreement with another report also using C57Bl/6 mice (Marathe et al., 2009) and suggest that PPARγ is dispensable for alternative activation per se. Although direct IL-4 responses are barely altered in PPARγ-deficient macrophages, STAT6 appears to be required for maximal PPARγ activation. Therefore, PPARγ might be more appropriately considered as a downstream effector in the hierarchy of IL-4-STAT6-PPARγ signaling. This scenario does not formally rule out that in vivo a liganded receptor can contribute to IL-4-regulated events in a more complex way. Therefore, the role of PPARγ in macrophages besides lipid handling remains to be mapped, whereas in human DCs it is linked to lipid metabolism and lipid antigen presentation (Szatmari et al., 2004, 2007).

IL-4 and PPARγ signaling appear to be connected at multiple levels. IL-4-mediated induction of *Pparg* and 12/15-lipoxygenase that could generate endogenous activators for PPARγ (Huang et al., 1999) provides two plausible mechanisms for enhanced response. Induction of *PPARg* itself is partly responsible for the enhancement, but IL-4-enhanced PPARγ activity appears much earlier than the lipoxygenase mRNA could be detected. Our results, presented here, point to STAT6 as the regulator of PPARγ response. Our global expression analyses showed that whereas STAT6 is required for maximal PPARγ response, PPARγ was largely dispensable for IL-4 signaling. This asymmetry is likely to be functionally important to provide specificity and allow STAT6 to act independently as well. The basis of this is an interaction by which STAT6 improves PPARγ activity via binding to the enhancer of PPARγ target genes. This is supported by three largely independent lines of evidence: the gene expression profile of STAT6-deficient macrophages, ChIP analysis of PPARγ and STAT6 target genes, and oligoprecipitation with the identified MacPPRE of *FABP4*. We propose therefore that STAT6 acts as a licensing factor for PPARγ to provide cell type-specific gene expression by enhancing DNA binding. Importantly, this interaction is specific; none of the other characterized receptors (PPARα, δ and LXRα) is influenced by STAT6. Additionally, probably as a special case of a robust IL-4-augmented PPARγ response, a conserved, complex enhancer (MacPPRE) exists in the *FABP4* gene. The mouse ortholog of this complex element behaves similarly to the human. We could detect PPARγ binding at both MacPPRE and AdipoPPRE in mouse adipocytes by analyzing recent ChIP-on-chip and ChIP-seq data (Lefterova et al., 2008; Nielsen et al., 2008). Although this complex enhancer has a binding site for STAT6 and contributes to the robustness of the response, it is not clear whether other genes also use bone fide STAT6 sites or rather protein-protein interactions for the enhancement. The demonstration that the two proteins can physically interact provides support for the latter. To explore this, we have made a model of the PPARγ:RXRα heterodimer (pdb3DZY) and the STAT6 dimer (based on the STAT3B-DNA complex [pdb1BG1]) on a B-form MacPPRE DNA element, which suggests that the two protein complexes sit in close proximity on the DNA (data not shown). Limited rearrangement of this complex could easily bring the proteins into direct physical contact. Alternatively, an indirect interaction might be mediated by corecruitment of a shared coregulator or even by the DNA acting as an allosteric effector, facilitating communication between the complexes.

A crosstalk between transcription factors in order to cooperatively orchestrate gene expression is not unprecedented. Estrogen receptor was reported to require the presence of another transcription factor, Forkhead box A1 (FoxA1), for efficient DNA binding and gene expression regulation (Carroll et al., 2005; Laganière et al., 2005). Recently, C/EBPs were reported to bind to the vicinity of PPARγ response elements in adipocytes (Lefterova et al., 2008; Nielsen et al., 2008). We suggest that STAT6 is likely to fulfill a similar role for PPARγ in macrophages and DCs. Although the molecular details of these crosstalks are still elusive, STAT6 might facilitate DNA binding of PPARγ or the two factors could synergistically recruit cofactors and chromatin remodeling enzymes. Alternatively, STAT6 itself might act as a coactivator to provide more efficient transactivation. It is intriguing to speculate that regulated and graded usage of licensing and facilitating factors (C/EBP and STAT6) could define specific responses of PPARγ leading to distinct gene expression programs in the various cell types or tissues.

## Experimental Procedures

### Materials

Ligands: LG268, LG1208, gifts from M. Leibowitz (Ligand Pharmaceuticals), WY14643, Rosiglitazone (RSG), T0901317, and MG132 (Alexis Biochemicals), GW501516, and GW9662 were gifts from T.M. Willson (GlaxoSmithKline). Cytokines were obtained from Peprotech. All other reagents were obtained from Sigma, consumables from Eppendorf, or as indicated.

### Isolation and Culture of Cells

Human monocytes were isolated from healthy volunteer's buffy coat, obtained with a Regional Ethical Board permit from the Regional Blood Bank, via CD14 MicroBeads (Miltenyi Biotec) and treated with vehicle (ethanol:dimethyl-sulfoxide 1:1) or as indicated. For activation we used IL-4 (100 ng/ml), IFN-γ (100 ng/ml), TNF (50 ng/ml), *E. coli* (O55:B5 serotype) LPS (100 ng/ml). Thioglycolate-elicited macrophages were harvested from the peritoneal cavity 4 days after injection of 3 ml 3% thioglycolate solution; bone marrow cells were isolated from the femur of mice. Mouse monocytes were isolated from bone marrow via negative selection method with magnetic separation (Miltenyi). Bone marrow cells were differentiated to macrophages by M-CSF (20 ng/ml) or to DCs by GM-CSF (20 ng/ml) and IL-4 (20 ng/ml) for 10 days. For activation we used mouse IL-4 (20 ng/ml), IFN-γ (20 ng/ml), TNF (20 ng/ml), *E. coli* (O55:B5 serotype) LPS (100 ng/ml).

### Real-Time Quantitative PCR

Total RNA was isolated with Trizol Reagent (Invitrogen). RNA was reverse transcribed with High Capacity cDNA Archive Kit (Applied Biosystems). Transcript quantification was performed by quantitative real-time PCR via Taqman probes. Transcript levels were normalized to cyclophilin A or 36B4. Details of primers are in Table S4.

### Immunoblotting

Total cell lysates or nuclear extracts were resolved in SDS-PAGE and immunoblotted with FABP4 (Cayman Chemical), GAPDH (Abcam), Flag (M2, Sigma-Aldrich), V5 (Serotech), PPARγ (E8), or STAT6 (M-20-Santa Cruz) antibodies as indicated.

### Transient Transfection

RAW264.7 cells were electroporated (300V for 15 ms); COS1 and HEK293T cells were transfected in triplicates with polyethyleneimine. Luciferase reporter activity was determined with Luciferase Assay System (Promega) and normalized to beta-galactosidase activity.

### Electrophoretic Mobility Shift Assay

PPARα, γ, δ and RXR were in vitro transcribed and translated with T7 Quick TNT Kit (Promega). DNA was labeled in a random priming reaction (Fermentas) with radioactive [^32^P]dATP. For competition, nonlabeled cold DNA (2–10×), for supershift experiments PPARγ (Perseus) or RXR (Perseus) antibodies were used.

### Pull-Down Assays

Human PPARγ1 was tagged with streptavidin-binding protein and expressed in Rosetta BL21 (Novagen). After induction with 40 μM isopropyl-D-thiogalactopyranoside, PPARγ1 was purified with streptavidin-resin. Whole cell lysates of STAT6 or mock-transfected HEK293T cells were added to the resin and after washing analyzed by immunoblotting.

### Coimmunoprecipitation

HEK293T cells were transfected with *STAT6-V5* and *Flag-PPARg* expression vectors. V5 (AB Serotech) or Flag M2 (Sigma) antibodies were used for immunoprecipitation and subsequent immunoblotting.

### Chromatin Immunoprecipitation

ChIP was performed as described earlier (Balint et al., 2005) with anti-PPARγ (CS-133-100, Diagenode and preimmune serum), anti-STAT6 (M20-Santa Cruz), or control immunoglobulin. Enrichment of genomic loci was quantitated with real-time PCR.

### Mice

Mice carrying null or floxed alleles of *Pparg* were described previously (Barak et al., 1999). These mice were bred with *LysCre* transgene animals obtained from I. Förster (Univ. of Munich) (Clausen et al., 1999). *Stat6*^−/−^ mice were purchased from The Jackson Laboratory. Animals were housed under minimal disease conditions and the experiments were carried out under institutional ethical guidelines and licenses.

### Microarray Analysis

Microarray analysis was performed with Affymetrix microarrays (Human Genome U133 Plus 2.0 or Mouse Genome 430 2.0) and standard protocols. Microarray hybridizations were carried out at the Debrecen Clinical Genomics Center Microarray Facility. Analysis was performed with GC-RMA on the cel files in GeneSpring 7.3 (Agilent). For each condition, three biological replicates were analyzed. Signal intensities were normalized to the 50^th^ percentile (per chip), then to the median expression of the certain gene throughout the experiment (per gene), and finally each chip was normalized to its specific vehicle-treated control. Changing genes were called based on a t test (parametric, variances assumed to be equal, with Benjamini and Hochberg false discovery rate), p < 0.05 and at least 1.5-fold changes. For annotation we used The Functional Annotation Tool at DAVID Bioinfomatics Resources 6.7 (National Institute of Allergy and Infectious Diseases [NIAID]).

### Immunohistochemistry

Immunostaining for PPARγ was carried out on paraffin-embedded cellular blocks with biotin-free Catalyzed Signal Amplification IHC detection kit (CSAII, Dako) and VIP substrate (Vector Labs). Sections were counterstained with methyl-green. Double immunofluorescence (IF) stainings were carried out on human tissues obtained from the archives of surgical tissue specimens of the Department of Pathology, University of Debrecen. For PPARγ staining, we used the red fluorescent tetramethyl-rhodamine (TMR)-tagged tyramide (Perkin-Elmer). All other IF for double stainings (CD68, DC-SIGN) were made sequentially via biotinylated secondary antibodies followed by a streptavidin-FITC development for green fluorescence (Dako). Sections were counterstained with 4',6-diamidino-2-phenylindole (DAPI, Vector Laboratories) (blue nuclear fluorescence).

### Bioinformatic Analysis

The PhastCons conservation scores for placental mammalian species were obtained from the UCSC site calculated from the MULTIZ (UCSC/Penn. State Bioinformatics) 44 vertebrate species whole-genome alignment.

### Oligoprecipitation Assays

Nuclear extracts from THP1 cells treated with vehicle or RSG+IL-4 were prepared as described earlier (Nagy et al., 2009). Precleared extracts were incubated with annealed biotin-labeled oligonucleotides representing MacPPRE and streptavidin-agarose. Captured protein was analyzed by immunoblotting with PPARγ (E-8) and STAT6 (M-20) antibodies (Santa Cruz).

### Statistical Tests

All data are presented as means ± SD and based on experiments performed at least in triplicate. Statistical tests were performed on the fold changes via unpaired (two tail) t test, p < 0.01.

## Accession Numbers

The microarray data are available in the Gene Expression Omnibus (GEO) database (http://www.ncbi.nlm.nih.gov/gds) under the accession number GSE16387.

## Supplemental Information

Document S1. Supplemental Experimental Procedures and Three Figures

Table S1. Genes Regulated in Human Macrophages by PPARγ Microarray experiment was performed on human nonactivated (C) or alternatively activated (IL-4 100 ng/ml) macrophages ± 1 μM RSG. Genes regulated after 12 hr at least 1.5-fold significantly (p < 0.05 with Benjamini-Hochberg multiple testing correction) are listed from Figures 1E and 1F. Subsets of up- and downregulated genes are listed in separate sheets. Functional annotation of RSG-regulated genes is also shown.

Table S2. Genes Regulated by IL-4 in Ppar*γ*^+/−^*LysCre* and *Pparg*^fl/−^*LysCre* Mouse Macrophages Microarray experiment was performed on mouse bone marrow-derived nonactivated (C) or alternatively activated (IL-4 20 ng/ml) macrophages ± 1 μM RSG from wild-type and *Pparg*^+/−^*LysCre* and *Pparg*^fl/−^*LysCre*. IL-4-regulated genes from Figures 2E and 2F (>2-fold, p < 0.05 with Benjamini-Hochberg multiple testing correction) are listed in separate sheets. Functional annotation of IL-4-regulated genes is also shown.

Table S3. Genes Regulated by PPARγ in Wild-Type and *Stat6*^−/−^ Mouse Macrophages Microarray experiment was performed on mouse bone marrow-derived noon-activated (C) or alternatively activated (IL-4 20 ng/ml) macrophages ± 1 μM RSG from wild-type and *Stat6*^−/−^ macrophages. RSG-regulated genes from Figures 3C and 3D (>2-fold, p < 0.05 with Benjamini-Hochberg multiple testing correction) are listed in separate sheets. Functional annotation of RSG-regulated genes is also shown.

Table S4. Real-Time PCR Assays Sequence of in-house designed Taqman primers and probes and catalog numbers of Taqman assays ordered from Applied Biosystems are listed.
