# Supplementary material for: STAT6 Transcription Factor Is a Facilitator of the Nuclear Receptor PPARγ-Regulated Gene Expression in Macrophages and Dendritic Cells
Source: Immunity. 2010 Nov 24;33(5):699–712. doi: 10.1016/j.immuni.2010.11.009 (PMC3052437; doi:10.1016/j.immuni.2010.11.009)
Supplement: Document S1. Supplemental Experimental Procedures and Three Figures [file mmc1.pdf]

Immunity, Volume *33*

Supplemental Data

STAT6 Transcription Factor Is a Facilitator of the Nuclear Receptor PPARγ-Regulated Gene Expression in Macrophages and Dendritic Cells

Attila Szanto, Balint L. Balint, Zsuzsanna S. Nagy, Endre Barta, Balazs Dezso, Attila Pap, Lajos Szeles, Szilard Poliska, Melinda Oros, Ronald M. Evans, Yaacov Barak, John Schwabe, and Laszlo Nagy

Szanto Supplementary Figure 1

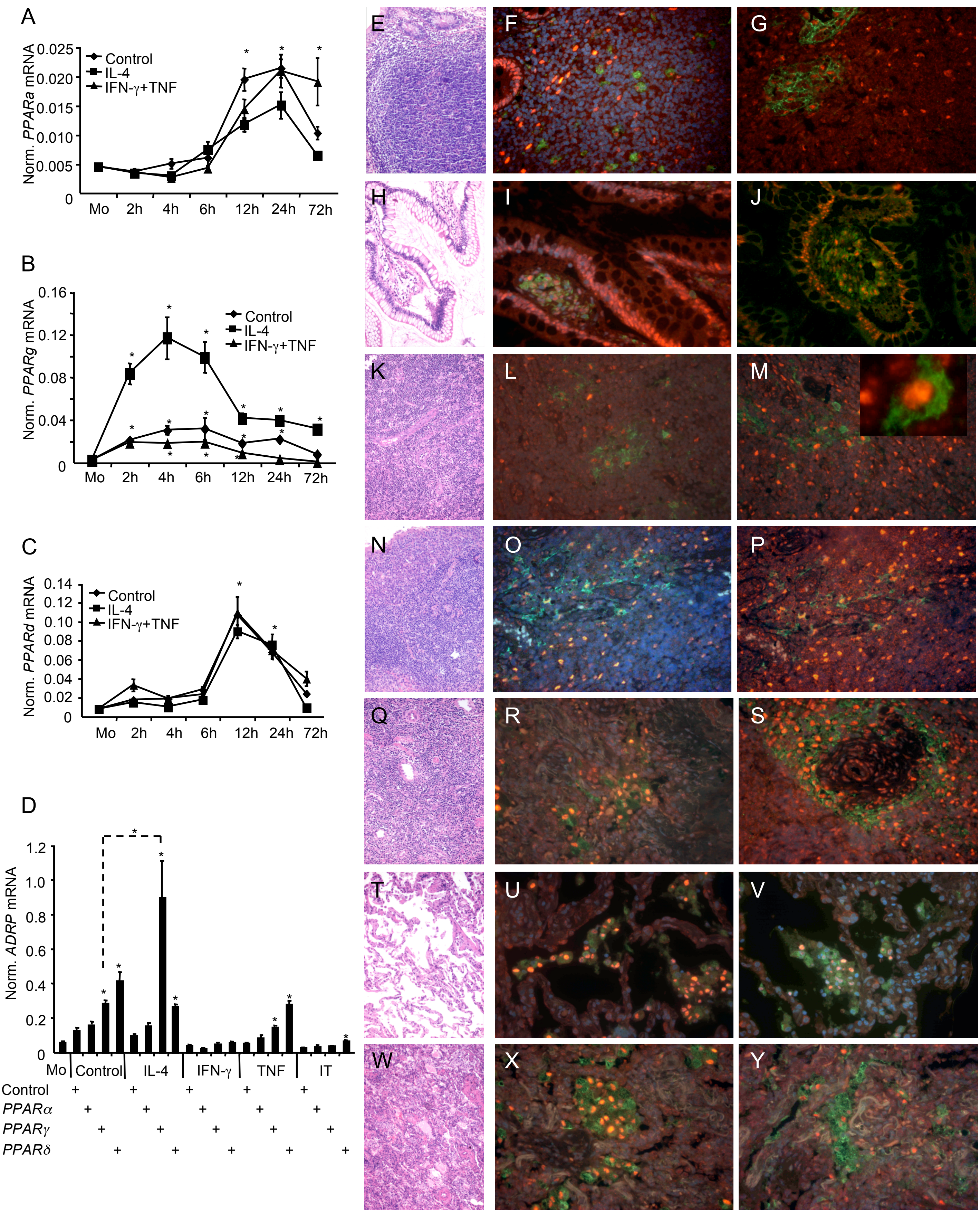

Figure S1. *PPARg* is specifically expressed in alternatively activated macrophages
(A-C) Human monocytes (Mo) were differentiated to macrophages for the indicated time. Macrophages were treated with vehicle (C), IL-4 (100 ng/ml), IFN-γ (100 ng/ml) + TNF (50 ng/ml). Expression of *PPARa* **(A)**, *PPARg* **(B)** and *PPARd* **(C)** was analyzed with real-time PCR.
(D) Human Mo-derived macrophages were cultured for 24h and treated with cytokines as in **(A-C)**. Additionally, cells were also treated with vehicle (DMSO:ethanol) (C) or activators of PPARα (10μM WY14643), PPARγ (1μM RSG) or PPARδ (1μM GW501516), respectively. Expression of *ADRP* was analyzed with real-time PCR. Transcript levels normalized to cyclophilin A are presented as means ±SD (n=3, *p<0.01).
(E-Y) Human lymphoid tissues were analysed such as Peyer's patches, lamina propria of the intestinal vili, reactive lymph nodes, tonsils and known areas of alternatively activated macrophages. CD68 was used as a reference marker for macrophages and DC-SIGN for alternatively activated macrophages and/or DCs (Geijtenbeek et al., 2000; Relloso et al., 2002). Our analysis suggested that (1) PPARγ was not expressed in each CD68 positive macrophage (middle panel: compare PPARγ positive red nucleated cells with CD68 positive cells having green cytoplasm), (2) PPARγ positive macrophages were not necessarily DC-SIGN positive (right panel: compare PPARγ positive red nucleated cells with DC-SIGN positive cells exhibiting green cytoplasm), but (3) almost every DC-SIGN positive macrophage expressed PPARγ (right panel). (4) PPARγ expressing macrophages/histiocytes were localized in compartments of the lymphoid organs, mainly around the germinal centers in the perifollicular T-cell areas (right panel, H). (5) PPARγ co-localized with DC-SIGN very prominently in perivascular and alveolar macrophages (left panel, O-U).
The following tissues are analyzed with double immunofluorescent staining: **(E-G)**: Peyer's patch and **(H-J)**: lamina propria of normal small intestinal villi, **(K-M)**: reactive lymph node, **(N-P)**: lymphoepithelial tissue of the tonsil, **(Q-S)**: perivascular macrophages of lymph node, **(T-V):** alveolar macrophages and **(W-Y):** T cell-mediated desquamative interstitial pneumonitis representing a chronic inflammatory disease of the lung. The left panels (*10x*) show hematoxylin-eosin-stained tissues that were used for immunofluorescent staining. The middle panels (*40x*) are double stained with anti-PPARγ-TMR (red nuclear fluorescence) and anti-CD68-FITC (green cytoplasmic fluorescence). The right panels (*40x*) are double stained with anti-PPARγ-TMR (red nuclear fluorescence) and anti-DC-SIGN-FITC (green cytoplasmic fluorescence). The insert in **(M)** shows the co-localization of PPARγ and DC-SIGN in a macrophage (*100x*).

Szanto Supplementary Figure 2

A

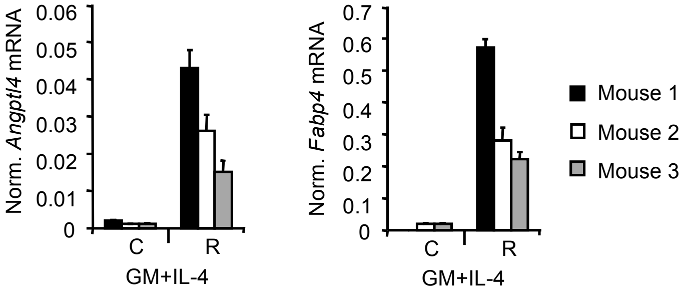

B

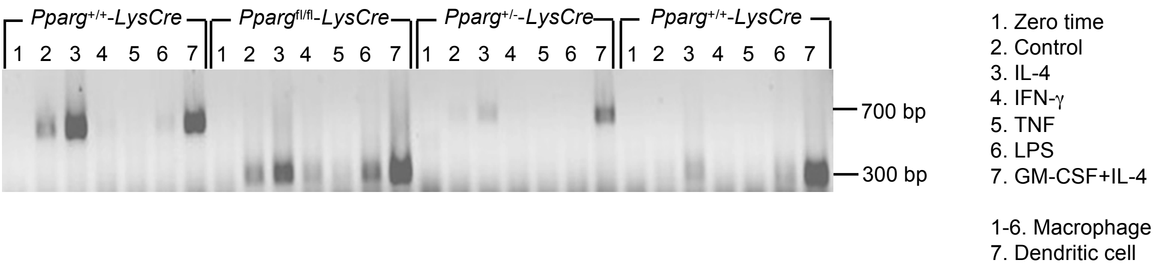

C

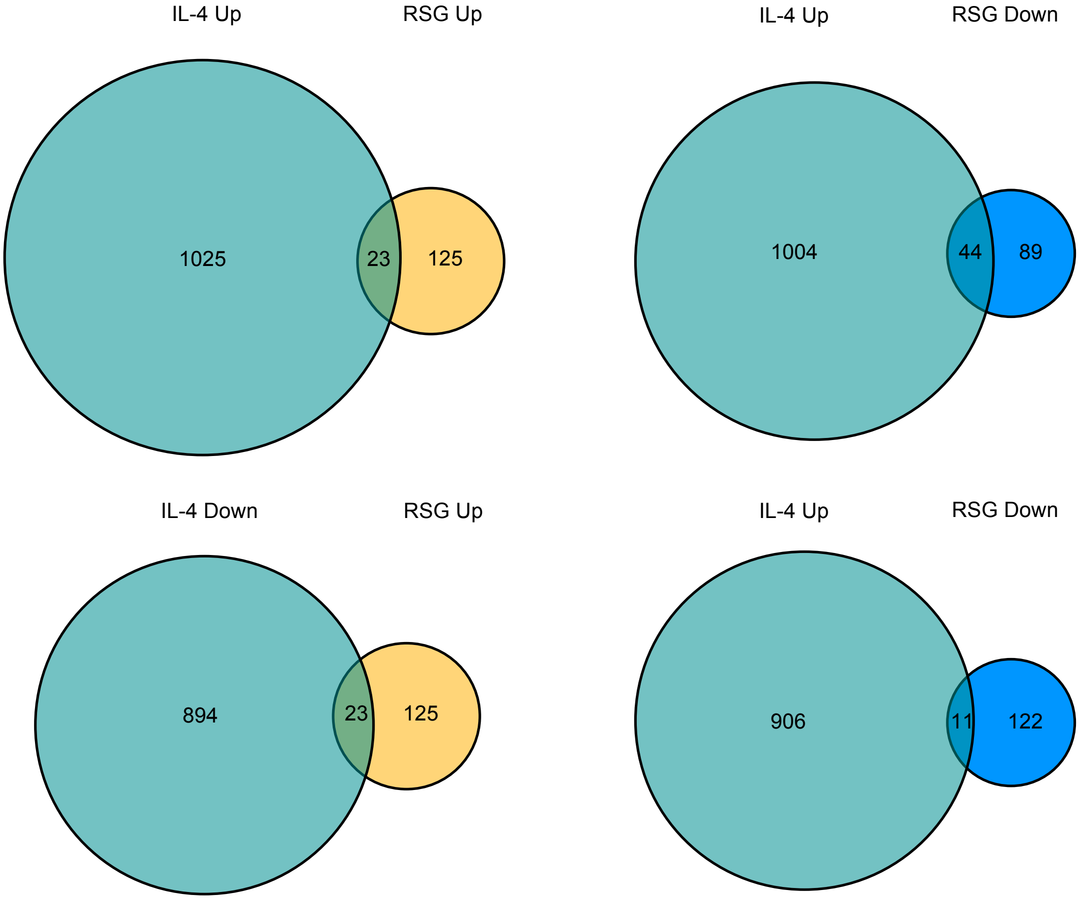

Figure S2. Expression of PPARγ target genes in mouse bone marrow-derived macrophages and DCs

(A) Mouse bone marrow-derived cells (n=3) were differentiated to immature DCs for 10 days in the presence of GM-CSF (20 ng/ml) + IL-4 (20 ng/ml) and treated with vehicle (C) or 1 μM RSG (R). Expression of *Angptl4* (left panel) and *Fabp4* (right panel) were determined by real-time PCR.

(B) Efficiency of recombination bone marrow-derived macrophages (20 ng/ml M-CSF), or DCs (20 ng/ml GM-CSF+20 ng/ml IL-4) of *Pparg* conditional knockout mice. Macrophages were activated by vehicle (C), IL-4 (20 ng/ml), IFN-γ (20 ng/ml), TNF (20 ng/ml) or LPS (100 ng/ml), respectively. Expression of wild-type (700 bp) or recombined (300 bp) *Pparγ* expression was analyzed with RT-PCR from cells isolated from *Pparg*$^{+/+}$-, *Pparg*$^{fl/fl}$-, *Pparg*$^{+/-}$- and *Pparg*$^{fl/-}$-*LysCre* mice.

(C) IL-4 and RSG-regulated genes were determined by microarrays generated from bone marrow-derived macrophages of *Pparg*$^{+/-}$ mice (n=3). Venn diagrams represent the overlap between IL-4-regulated and RSG-regulated genes. Up and downregulated genes are shown separately. The numbers indicate that only a minor part of IL-4-regulated genes are also regulated by PPARγ with no preferential correlation between the upregulated or downregulated genes.

Szanto Supplementary Figure 3

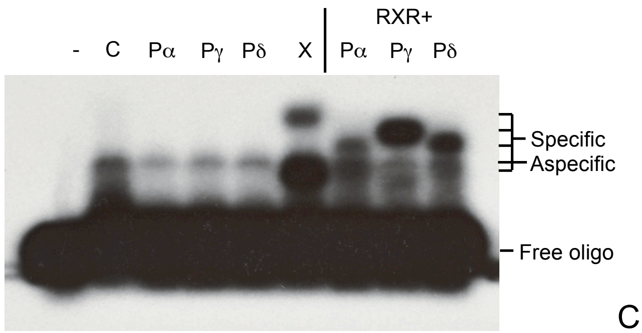

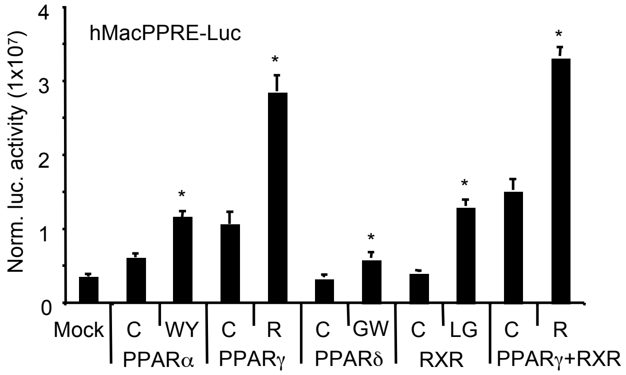

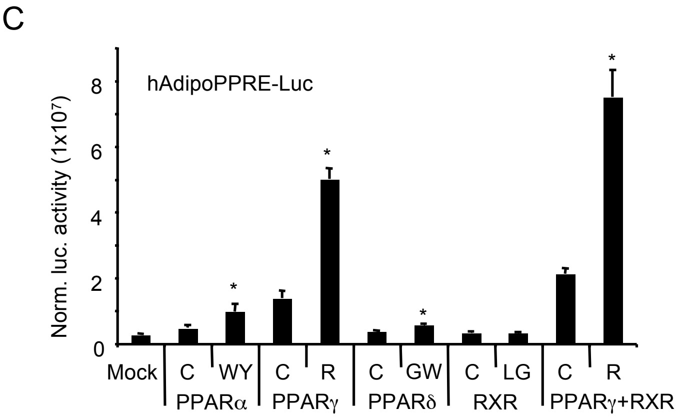

D

MacPPRE **STAT6**

```
         10        20        30        40        50        60        70        80        90       100
                               ----:----|----:----|----:----|----:----|----:----|----:----|----:----|----:----|----:----|----:----|
Consensus     cctcatttcctcnncaaaagaaataaacaCAGGCAaAGGTCAGagggangcaTTCcatgGAAgctgtccatagnagagcaaagtgcaaggcagacggcct
Human         tCTCATTTCCTC--CAAAAGAAATAAACACAGGCAAAGGTCAGAGGGAtGCATTCCATGGAAGCTGTCCATAGgAGAGCAAAGTGCAAGGCAGACGGCCT
Chimp         tCTCATTTCCTC--CAAAAGAAATAAACACAGGCAAAGGTCAGAGGGAcGCATTCCATGGAAGCTGTCCATAGgAGAGCAAAGTGCAAGGCAGACGGCCT
Marmoset      tCTCATTTCCTC--CAAAAGAAATAAACACAGGCAAAGGTCAGAGGGAcGCATTCCATGGAAGCTGTCCATAGgAGAGCAAAGTGCAAGGCAGACGGCCT
Gorilla       tCTCATTTCCTC--CAAAAGAAATAAACACAGGCAAAGGTCAGAGGGAcGCATTCCATGGAAGCTGTCCATAGgAGAGCAAAGTGCAAGGCAGACGGgCT
Orangutan     tCTCATTTCCTC--CAAAAGAAATAAACACAGGCAAAGGTCAGAGGGAcGCATTCCATGGAAGCTGTCCATAGgAGAGCAAAGTGCAAGGCAGAtGGCCT
Rhesus        CCTCATTTCCTC--CAAAAGAAATAAACACAGGCAAAGGTCAGAGGGAtGCATTCCATGGAAGCTaTCCATAGgAGAGtAAAGTGCAAGGCAGAtGGCCT
Squirrel      CCTCATTTCCTC--CAAAAGAAATAAACACAGGCAAAGGTCAGAGGGAtGCATTCaATGGAAGCTGcCCATAGgAGAGCAAAGTGCAAGGCAGACaGCCT
Bushbaby      CCTCATTTCCTC--CAAAAGAAATAAACACAGGCAAAGGTCAGAGGGAtGCATTCCATGGACGCTGTCCATAGaAGAGCAAAGTGCAAGGCgGAgGGCCg
Dog           CCTCATTTCCTC--CAAAAGAAATAAACACAGGCAAAGGTCAGAGGGAtGCATTCCATGGAAGCTGTCCATAGtgcAaCAAAGTGCAAaGCAGt-GGCCT
Tarsier       CCTCATTTCCTt--tAAAAGAAATAAACACAGGCAAAGGTCAGAGGGAcGCATTCCATGGAAGCTGTCCATAGgAGAGCAAAGTGCAgGGCAagCaGCCT
Cat           CtTCATTTCCTC--CAAAAGAAATAAACAAAGGCAAAGTTCAGAGGGAaGCATTCCATGGAAGCTGTCCATAGtAGAGCAAAGTGCAAaGCAatCaGCCT
Microbat      CCTCATTTCCTC--CAAAAGAAATAAACACAGGCAAAGGTCAGgGGGAcGCATTCCATGGACGCTGTCCAcAGtgGAGCAAAGTGCAg-GCAGgCGG-CT
Mouse lemur   CCTaATTTCCTC--CgAAAGAAAcAAACACAGGCGAAGGTCAGAGGGAcaCATTCCATGGACGCTGTCCATAGgAGAGCAAAGTGCAAGGCAGAgGGCCc
Horse         aCTCATTTCCTC--tAAtAGAAATAAACACAGGCAAAGGTCAGAGGGAtGCATTCCATGGCAGCTGcCCATAGtAGAGCAAAGTGCgAGGCAGgCGGtCT
Hedgehog      CtTCATTTCCTC--CAAAAGAAATAAACACAGGCAAAGGTCAAAGGGAtGCATTCCATGGACatTGTCCAgAGgAGAGCAAAGTGCAAGGCAGgCaGCCT
Dolphin       gCTCATTTCCTC--CAAAAGAAATAAACACAGGCAAAGGTCATAGGGAtaCATTCCATGGAAGCTGTCCATcatAGAGCAAAGTGCAAaGCAGctGGtCT
Cow           CCTgATTTCCTC--CAAAAGAAATAAgCAGAGACAAAGGTCAGAGGGAtaCATTCCATGGAAGCTGTCCATcGtAGAGCAAAGTGCAAaGCAGctGGCCT
TreeShrew     CCTaATTTCCTC--CAAAAGAAATAAACACAGGCAAAGGTCAGAGGGAcaCATTCCgTGGAAGCTGTCCATAGgAGAGCAAAGTGCgAGGCAaAgtGttc
Rabbit        CCTCATTTCCTt--CAAAAGAAATAAACACAGACAAAGGTCAGAGGGAcGCATTCCATGGATGCTGTCCATAGgAGAGCAAAGTGCAgaaCtaAtaGCCT
Shrew         CCTCATTTCCTC--CcAAAGAAATAAACACAGGCAAAGTTCAGcGGGAcGCATTCCATGGAAGCTGgCCAcAGtAGAGCAAAGTGCAgaGCtGtCaGCCT
Kangaroo rat  CCTCATTTCCTC--CcAAAGAAATAAACACAGACAAAGGTCAGgGGaAtGCATTCCATGGACaCTGTCCATAGtgGAGCAAAGTGCAtGGCAagCaGCCT
Sloth         CCTCATTTCCTC--CAAAAaAAATAAACACAGGCAAAGGTCAGAGaGccaCATTCCATGGATaCTGTCCAcAGtgGAGCAAAGTGCAAGGCAcAgGGCCa
Rat           tgTCATTTCCTC--CAAAAGAAATAAACACAGGCAAAGGTTAGAaGGAtGCATTCCATaGAGGCTGcCCATttagGAGCAAAGTGCAAGGCAaACaGCCT
Mouse         tCTCATTTCCTC--CgAAAGAAgTAAACACAGGCAAAGGTTAGAaGGAtGCATTCCAcaGAAaCgGcCCATtGagGAGCAAAGTGCAAGGCAaACaGCCT
Pika          CCTCATTTCCTt--CAAAAcAAAcAgACACAGACAAAGGTCAGAGGGAcGCATTCCATGGATGCTGTCCATgGgAGAGCAAAGTGCAgaGCtagCatCCT
Guinea Pig    tCTCAcTTCCTC--CAAAAGAAATAAACACGGGAAAAGGTCAGcaGGAgGCATTCCATGGAAGCTacCCAgAGgAcAGCAAAGTGCgcGGCcagCaGtCc
Elephant      ttTCcTTTttct--CAAAAGAAAcAAACACGGGCAAAGGTCAGAGGGAtGCATTCCATGGAAGCcaTCCATAGtgGAGCAAAGTGCAAaGCAaACcaCtg
Armadillo     CCTCATTTCCTtaatAAAAtAAAatAgtACAGGCAAAGGTCAGAGaGc-GCATTCCATGGCCaCTGTCCAcAGtAGAGgAAAGTGCAAaaaA-AtGGCCT
Alpaca        tCctATTTCCTC--CAAAAGAAATAAACtCAGACAAAGGTCAGAGtcAtGtgTTCCATGGAAcCTGctCgctGtAGAGCAAAGgGCgAGGCAactGGCtT

Consensus     cctcatttcctcnncaaaagaaataaacaCAGGCAaAGGTCAGagggangcaTTCcatgGAAgctgtccatagnagagcaaagtgcaaggcagacggcct
```

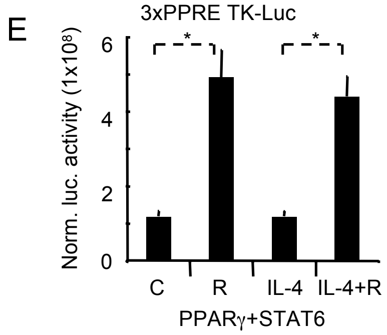

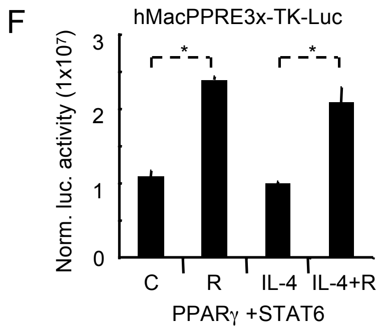

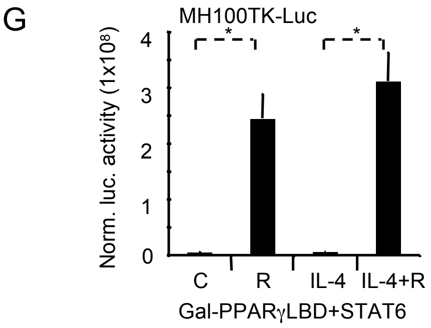

Figure S3. Analysis of PPARγ binding site in the promoter of *FABP4*

(A) 1st halfsite of the human MacPPRE was mutated to the consensus AGGTCA and binding of PPARα (Pα), PPARγ (Pγ), PPARδ (Pδ), RXR (X) and the heterodimers were analyzed with EMSA.

(B-C) COS1 cells were transfected with reporter constructs containing human MacPPRE **(B)** or AdipoPPRE **(C)** and mock, *PPARa*, *PPARg*, *PPARd*, *RXRa* and *PPARg+RXRa* expression vectors as indicated. Cells were treated with vehicle (C) or activators of PPARα (10μM WY14643 – WY), γ (1 μM RSG – R), δ (1 μM GW501516 – GW) or RXR (100 nM LG268 – LG) respectively. Normalized reporter activity is presented as means ±SD (n=3, *p<0.01).

(D) Promoter sequences of 29 mammals were compared and the result of alignment is shown.

(E-F) 3 copies of isolated consensus **(E)** or hMacPPRE **(F)** were cloned into reporter vector and cotransfected with *PPARg* and *STAT6* expression vectors into 293T cells. Luciferase activity was measured 24h after ligand treatment (vehicle (C) or 1 μM RSG (R), 100 ng/ml IL-4 or IL-4+RSG). Normalized reporter activity is presented as means ±SD (n=3, *p<0.01).

(G) 293T cells were transfected with *Gal-PPARg* ligand-binding domain, *STAT6* and MH100TK-Luciferase reporter construct containing Gal binding sites. Cells were treated with vehicle (C), 1 μM RSG (R), 100 ng/ml IL-4 or IL-4+RSG for 24h. Normalized reporter activity is presented as means ±SD (n=3, *p<0.01).

Supplementary experimental procedures

Isolation and culture of human monocytes

Human monocytes were isolated from healthy volunteer's buffy coat obtained from the Regional Blood Bank. Separation was carried out according to the manufacturer's instructions using CD14 MicroBeads (Miltenyi Biotec). Monocytes were differentiated for the indicated time. Cells were cultured in RPMI1640 supplemented with 10% FBS, 2mM glutamine, penicillin and streptomycin and treated with vehicle (ethanol:dimethyl-sulfoxide 1:1) or as indicated. For the activation we used IL-4 (100 ng/ml), IFN-γ (100 ng/ml) and TNF (50 ng/ml), *E. coli* (O55:B5 serotype) LPS (100 ng/ml). The concentrations of inhibitors used in the experiments are as follows: MG132 (10 μM), JAK3 inhibitor (WHI-P131) (80 μM), JAK2 inhibitor TyrPhostin AGN490 (TyrPh.) (80 μM) or PI-3K inhibitor, wortmannin (200 nM).

Isolation and culture of mouse peritoneal and bone marrow cells

Thioglycolate-elicited macrophages were harvested from the peritoneal cavity 4 days after injection of 3 ml 3% thioglycolate solution. Cells were washed in saline and cultured in RPMI1640 supplemented with 10% FBS, 2 mM glutamine, penicillin and streptomycin for two days. Bone marrow cells were isolated from the femur of mice then were washed in saline; cultured in RPMI1640 supplemented with 10% FBS, 2mM glutamine, penicillin and streptomycin. Monocytes were isolated from bone marrow using negative selection method with magnetic separation (Miltenyi). Bone marrow cells were differentiated to macrophages by M-CSF (20 ng/ml) or to DCs by GM-CSF (20

ng/ml) and IL-4 (20 ng/ml) for 10 days. For activation of macrophages similar methods were used as in case of the human macrophages: IL-4 (20 ng/ml), IFN-γ (100 ng/ml), TNF (20 ng/ml), *E. coli* (O55:B5 serotype) LPS (100 ng/ml). Fresh medium containing cytokines and ligands were added every third day to complement the old medium.

RNA isolation and real-time quantitative RT PCR

Total RNA was isolated from cells using Trizol Reagent (Invitrogen) according to the instructions. RNA was transcribed into cDNA via random hexamer priming using SuperScript II (Invitrogen) reverse transcriptase or High Capacity cDNA Archive Kit (Applied Biosystems). Transcript quantification was performed by quantitative real-time RT (reverse transcriptase) PCR (polymerase chain reaction) using Taqman probes (self-made assays) or Taqman Gene Expression Assays (Applied Biosystems). Transcript levels were normalized to the level of cyclophilin A. Sequences of primers and Taqman probes or Taqman Assays IDs used in transcript quantification are listed in Suppl. Table 4.

Western blotting

Cells were treated for two days as indicated and were washed in PBS then lysed in buffer A (100 mM Tris-HCl pH 7.5, 1 mM EDTA, 15 mM β-mercaptoethanol, 0.1% Triton X 100, 0.5 mM PMSF (phenyl-methyl-sulfonyl fluoride). 25 μg total protein was separated on 10% SDS-PAGE (polyacrylamid gel electrophoresis) and transferred to PVDF membrane (Millipore). Similarly, nuclear extracts were resolved in SDS-PAGE and blotted. After blocking with 5% dry milk the membrane was probed with anti-FABP4

(Cayman Chemical), anti-GAPDH (Abcam), anti-Flag (M2, Sigma-Aldrich), anti-V5 (Serotech), anti-PPARγ (E8) or anti-STAT6 (M-20-Santa Cruz) antibodies as indicated. Peroxidase-conjugated secondary antibody and ECL detection kit (Pierce) was used for signal detection.

Transient transfection

For transient transfection experiments COS1, HEK293T or RAW264.7 cells were used as indicated. RAW264.7 cell were electroporated with vectors using one square-wave impulse of 300V for 15 ms. COS1 and HEK293T cells were transfected with polyethyleneimine in 48-well format using triplicates. 2 days after transfection cell were lysed and analyzed by immunoblotting, quantitative RT PCR, pull-down experiments or reporter-activity was determined according to the protocol of the Luciferase Assay System (Promega). Reporter activity was normalized to beta-galactosidase activity.

Electrophoretic mobility shift assay (EMSA)

PPARα, γ, δ and RXR were *in vitro* transcribed/translated using T7 Quick TNT Kit (Promega). DNA was labeled in a random priming reaction (Fermentas) with radioactive ^{32}P-dATP and was added to the *in vitro* translated proteins in a binding buffer (20 mM Hepes pH 7.9, 50 mM KCl, 0.1 mM EDTA, 1 mM dithiothreitol, 1 mM AEBSF, 1.5 µg poly(deoxyinosinic-deoxycytidylic) and 5% glycerol. For competition non-labeled cold DNA (2-10x) was added prior to the reaction. For supershift experiments anti-PPARγ (Perseus) or anti-RXR (Perseus) antibodies were used. After 30 min DNA-protein complexes were separated in 0.7% agarose gel in 0.5x Tris-borate buffer. Gels were dried

and radiography was performed to detect the DNA. The following oligonucleotides were used in binding reactions:

DR1: tcgacTCGAGGGTAGGGGTCAGAGGTCACTCGTg

haP2 PPRE: tcgacAATAAACACAGGCAAAGGTCAGAGGGATGCATTCCAg

haP2 PPRE M1: tcgacAATAAACAccaaggAAGGTCAGAGGGATGCATTCCAg

haP2 PPRE M2: tcgacAATAAACACAGGCAAatcggGAGGGATGCATTCCAg

haP2 PPRE M3: tcgacAATAAACACAGGCAAAGGTCAGtaagggGCATTCCAg

haP2 PPRE Cons: tcgacAATAAACAaggtcaAAGGTCAGAGGGATGCATTCCAg

Pull-down assays

Human *PPARγ1* cDNA fused to a streptavidin-binding protein and to a calmodulin-binding protein was cloned into a modified pET30a vector and was expressed in Rosetta BL21 (Novagen) after overnight induction with 40 μM isopropyl-D-thiogalactopyranoside (IPTG). Bacteria were lysed in non-denaturing lysis buffer with 3 freeze-thaw cycles and 5 minutes sonication. PPARγ1 was purified with streptavidin-resin. Whole cell lysates of STAT6- or mock-transfected HEK293T cells were added to the resin and incubated overnight. After 3 washing steps samples were boiled in Laemmli buffer and analyzed by immunoblotting.

Co-immunoprecipitation

HEK293T cells were transfected with the indicated expression vectors using polyethyleneimine and lysed after 2 days in non-denaturing lysis buffer (50 mM Tris-HCl pH 7.9, 100 mM KCl, 5mM MgCl2, 10% glycerol, 1 mM 4-(2-Aminoethyl)

benzenesulfonyl fluoride hydrochloride (AEBSF), 25 μM p-nitrophenyl-p′-guaninebenzoate (pNPGB), 1 mM dithiothreitol, 0.1% NP-40, protease inhibitor cocktail). Anti-V5 (AB Serotech) or anti-Flag M2 (Sigma) antibodies were used for immunoprecipitation overnight, then Protein G (Pierce) was added for 3 hours. Beads were washed in lysis buffer 3 times and samples were boiled in Laemmli-buffer.

Chromatin immunoprecipitation

Chromatin immunoprecipitation was performed as described earlier (Balint et al., 2005) with modifications. Briefly, cells were fixed with 1% formaldehyde for 10 minutes, nuclei isolated and lysed in FA buffer (50 mM Hepes, 140 mM NaCl, 1 mM EDTA, 1% Triton X-100, 0.1% sodium deoxycholate, 0.1 % SDS). Immunoprecipitation was performed overnight with the antibodies against PPARγ (pAb-133-050-Diagenode and preimmune serum), STAT6 (M20-Santa Cruz) or control immunoglobulin. Precipitates were bound to magnetic Protein A and washed. Beads were washed five times with FA buffer and DNA purified with Chelex (Biorad). Real-time PCR assays for the genomic loci were performed with Applied Biosystem Minor Groove Binding Taqman assays or the Roche UPL system. Oligonucleotides used are available upon request.

Mice

Mice carrying null or floxed alleles of *Pparg* were created as described previously (Barak et al., 1999; He et al., 2003; Hevener et al., 2003; Miles et al., 2000). These mice were backcrossed to the C57BL/6J strain for eight generations. Mice were bred with lysozyme-Cre (*LysCre*) transgene animals to create the following genotypes: *Pparg*$^{+/+}$ *LysCre*,

Pparg$^{fl/fl}$ *LysCre*, *Pparg*$^{+/-}$ *LysCre* and *Pparg*$^{fl/-}$ *LysCre*. Genotypes were determined by PCR of tail DNA. PCR genotyping was carried out by using the following primers: for the *Cre* transgene, 5'-GCATTACCGGTCGATGCAACGAGTG-3' and 5'-GAACGCTAGAGCCTGTTTTGCACGTTC-3'; for the upstream loxP site, 5'-CTAGTGAAGTATACTATACTCTGTGCAGCC-3' and 5'-GTGTCATAATAAACATGGGAGCATAGAAGC-3'; and for the null allel, 5'-AGGCCACCATGGAAAGCCACAGTTCCTC-3' and 5'-GCTGGCGAAAGGGGGATGTGCTGCAAG-3'. Genomic DNA was amplified by 35 cycles of 94°C for 20 s, 60°C for 30 s, and 72°C for 55 s.

RT PCR was performed with SuperScript II (Invitrogen). Sense (5'-GTCACGTTCTGACAGGACTGTGTGAC-3') and antisense (5'-TATCACTGGAGATCTCCGCCAACAGC-3') primers were designed to anneal to regions in exons A1 and 4 of *Pparg1*, respectively, which distinguish the full-length (700-bp) and recombined (300-bp) transcripts (He et al., 2003). PCR was performed by 40 cycles of 94°C for 20 s, 60°C for 30 s, and 72°C for 60s. *LysCre* animals were obtained from I. Förster (University of Munich) (Clausen et al., 1999). *Stat6*$^{-/-}$ mice were purchased from The Jackson Laboratory. All animal experiments were carried out under ethical guidelines, which were established by the 28th Act in 1998 of the Parliament of the Republic of Hungary. Animals were housed under minimal disease *(MD)* conditions in a laboratory animal facility seeing the requirements of FELASA recommendations and DIN EN ISO 9001 standards. Animal boxes were ventilated with HEPA filtered air, animals received sterilized pellet diet (Altromin) and tap water (*ad libitum).* The cages contained sterilized bedding material. The room lightning was automatically switched on

at 6:00 and off at 18:00. The room temperature was 20± 2 °C, the relative humidity was 50%.

Microarray analysis

Microarray analysis was performed on human and mouse macrophages. We used wild-type, *Pparg*$^{+/-}$*LysCre*, *Pparg*$^{fl/-}$*LysCre* and *Stat6*$^{-/-}$ mouse macrophages and analyzed them on Affymetrix microarrays. Total RNA was isolated using RNeasy kit (Qiagen). cRNA was generated from 5 µg of total RNA by using the SuperScript Choice Kit (Invitrogen) and the High Yield RNA transcription labeling kit (Enzo Diagnostics). Fragmented cRNA was hybridized to Affymetrix arrays (Human Genome U133 Plus 2.0 or Mouse Genome 430 2.0) according to Affymetrix standard protocols. Analysis was performed using GeneSpring 7.3 (Agilent). The Affymetrix cel files were loaded into GeneSpring and analyzed by GC-RMA. In case of human microarrays 3 biological replicates (3 for each condition, non-activated and alternatively activated plus/minus RSG) of the 12h-treated samples were analyzed. Similarly, samples from 3 *Pparg*$^{+/-}$*LysCre* and 3 *Pparg*$^{fl/-}$*LysCre* mice or 3 wild-type C57Bl/6 and 3 *Stat6*$^{-/-}$ mice were analyzed, resepectively. Non-activated or IL-4-activated mouse bone marrow-derived macrophages were treated with vehicle or 1 µM RSG. After per chip normalization to the 50th percentile of expression values obtained from the whole array we performed per gene normalization to the median expression of the given gene during the various conditions and finally each chip were normalized to its specific vehicle-treated control. After determining the changing genes based on a T-test (parametric, variances assumed to be equal, using Benjamini and Hochberg false discovery rate as multiple testing correction), p-value

cutoff 0.05 we selected those genes that showed at least 1.5 (human) or 2-fold (mouse) changes. All microarray data were deposited in GEO (GSE16387). For the bioinformatical analysis and functional annotation of gene lists we used The Functional Annotation Tool at DAVID Bioinfomatics Resources 6.7 (National Institute of Allergy and Infectious Diseases (NIAID), NIH: http://david.abcc.ncifcrf.gov/home.jsp.

Immunohistochemistry

For immunocytochemistry, macrophages ($6x10^6$ cells/group) were pelleted and fixed in 4% paraformaldehyde (pH 7.3) for 24 h at 4^oC. Cell blocks were then embedded in paraffin followed by serial sectionings (4 μm thick). After deparaffinisation and dehydration, serial sections from each cell group, mounted on the same glass slides, were used for peroxidase-based indirect immunohistochemistry (IHC). Briefly, sections were treated with 3% H_2O_2 in methanol for 15 min at room temperature to block the endogenous peroxidase. For antigen unmasking, sections were heated in antigen retrieving citrate buffer (pH 6.0, Dako) for 2 min at 120^oC using a pressure cooker. Immunostainings of cells for PPARγ were carried out using the biotin-free Catalyzed Signal Amplification IHC detection kit according to the manufacturer's instructions (CSAII, Dako). After blocking the non-specific binding sites, sections were incubated with the primary antibodies for 1h at room temperature prior to use the secondary antibodies. The peroxidase-mediated color development was set up for 5 min using the VIP substrate (Vector Labs). Finally, the sections were counterstained with methyl-green. Double immunofluorescence (IF) stainings were carried out on human tissues obtained from the archives of formalin-fixed and paraffin-embedded surgical tissue specimens of

the Department of Pathology, University of Debrecen. Following the incubations with the primary then the HRP-conjugated secondary goat anti-mouse antibodies, IF for PPARγ staining was carried out using the red fluorescent tetramethyl-rhodamine (TMR)-tagged tyramide reagent of the fluorescent amplification kit according to the manufacturer's instructions (Perkin-Elmer). After rinsing, all other IF for double stainings (CD68, DC-SIGN) were then made sequentially using biotinylated secondary antibodies in complexes followed by a streptavidin-FITC development for green fluorescence (Dako). After rinsing, the sections were counterstained with 4',6-diamidino-2-phenylindole (DAPI, Vector Laboratories) (blue nuclear fluorescence). Fluorescent microphotographs were made with single exposure using excitation filter to visualize simultaneously green (FITC), red (TMR), together with the blue (DAPI) fluorescence lights.

Bioinformatic analysis

The PhastCons conservation scores for placental mammalian species were obtained from the UCSC site. The scores were calculated from the MULTIZ (UCSC/Penn State Bioinformatics) 44 vertebrate species whole genome alignment. The graph in Figure 5G shows the 10kbp promoter region of the human *FABP4* gene until the second exon. It corresponds to the hg18 chromosome 8 genomic position from 82555216 to 82568028 on the negative strand. The X-axis shows the (decreasing) chromosomal position on the negative strand. The Y-axis shows the PhastCons conservation scores (in the range 0-1) for each position. The wigFix PhastScore files were converted with a PERL script to a bed-file style comma-separated format suitable for spreadsheet processing. The empty (without PhastScore) regions were filled with zeros.

Oligoprecipitation Assays

Biotin-labeled hMacPPRE (wild-type (WT) sense: tccaaaagaaataaacacaggcaaaggtcagagggatgcattccatggaagctgtccataggagagca, antisense: tgctctcctatggacagcttccatggaatgcatccctctgacctttgcctgtgtttatttcttttgga), its mutant counterparts (PPRE mutant (M-PPRE) sense: tccaaaagaaataaaca**ccaaggaaatcgg**gagggatgcattccatggaagctgtccataggagagca, antisense: tgctctcctatggacagcttccatggaatgcatccctc**ccgatttccttgg**tgtttatttcttttgga, STAT6 binding site mutant (M-S6) sense: tccaaaagaaataaacacaggcaaaggtcagagggatgca**tcccatggag**gctgtccataggagagca, antisense: tgctctcctatggacagc**ctccatggga**tgcatccctctgacctttgcctgtgtttatttcttttgga and double mutant (M-PPRE-S6) sense: tccaaaagaaataaaca**ccaaggaaatcgg**gagggatgca**tcccatggag**gctgtccataggagagca, antisense: tgctctcctatggacagc**ctccatggga**tgcatccctc**ccgatttccttgg**tgtttatttcttttgga) or scrambled control oligonucleotides (sense: gatacgaacatgacgatgcacaatagagatgcgaacatagcgatgcgacatagacatgcgaagatagc, antisense: gctatcttcgcatgtctatgtcgcatcgctatgttcgcatctctattgtgcatcgtcatgttcgtatc) were annealed in buffer A (5x: 1M Tris pH7.6, 1M $MgCl_2$, 1M DTT) by heating to 95 °C then slowly cooling to room temperature. THP1 cells were treated with vehicle (DMSO:ethanol) for 1h or 1 μM RSG for 30 min and 20 ng/ml IL-4 for an additional 30 min. Then nuclear extracts prepared as described earlier (Nagy et al., 2009). Nuclear extracts were diluted 2-fold with OP buffer (10 mM HEPES pH7.9, 1.5 mM $MgCl_2$, 10 mM KCl, 0.5 mM DTT, 1 mM sodium-orthovanadate and 0.4% NP-40) then pre-cleared with 10 μg scrambled

control oligonucleotide in the presence of 20 μg salmon sperm DNA, 10 μg poly(dIdC) and 50 μl streptavidin coupled agarose beads (Sigma, 50% slurry) for 30 min at 4 °C. Pre-cleared extracts were incubated with 10 μg MacPPRE and 50 μl streptavidin-agarose for 2h at 4 °C, and then washed three times with OP buffer. Captured protein was resolved by SDS-PAGE (7.5%) and Western blotted with monoclonal anti-PPARγ antibody (E-8, Santa Cruz). The membrane was then stripped and re-probed with STAT6 antibody (M-20, Santa Cruz)

Statistical tests

All data are presented as means ±SD. In real-time quantitative PCR and reporter assays the mean and standard deviation were calculated for both the normalized and the normalizer values. To incorporate the random errors of the measurements we used the propagation of errors to determine the standard deviation of the normalized values. For all experiments we made at least three biological replicates and on the fold changes we performed an unpaired (two tail) t test and results were considered significant with $p<0.01$.

Supplementary references

Balint, B. L., Szanto, A., Madi, A., Bauer, U. M., Gabor, P., Benko, S., Puskas, L. G., Davies, P. J., and Nagy, L. (2005). Arginine methylation provides epigenetic transcription memory for retinoid-induced differentiation in myeloid cells. Mol Cell Biol *25*, 5648-5663.

Barak, Y., Nelson, M. C., Ong, E. S., Jones, Y. Z., Ruiz-Lozano, P., Chien, K. R., Koder, A., and Evans, R. M. (1999). PPAR gamma is required for placental, cardiac, and adipose tissue development. Mol Cell *4*, 585-595.

Clausen, B. E., Burkhardt, C., Reith, W., Renkawitz, R., and Forster, I. (1999). Conditional gene targeting in macrophages and granulocytes using LysMcre mice. Transgenic Res *8*, 265-277.

Geijtenbeek, T. B., Torensma, R., van Vliet, S. J., van Duijnhoven, G. C., Adema, G. J., van Kooyk, Y., and Figdor, C. G. (2000). Identification of DC-SIGN, a novel dendritic cell-specific ICAM-3 receptor that supports primary immune responses. Cell *100*, 575-585.

He, W., Barak, Y., Hevener, A., Olson, P., Liao, D., Le, J., Nelson, M., Ong, E., Olefsky, J. M., and Evans, R. M. (2003). Adipose-specific peroxisome proliferator-activated receptor gamma knockout causes insulin resistance in fat and liver but not in muscle. Proc Natl Acad Sci U S A *100*, 15712-15717.

Hevener, A. L., He, W., Barak, Y., Le, J., Bandyopadhyay, G., Olson, P., Wilkes, J., Evans, R. M., and Olefsky, J. (2003). Muscle-specific Pparg deletion causes insulin resistance. Nat Med *9*, 1491-1497.

Miles, P. D., Barak, Y., He, W., Evans, R. M., and Olefsky, J. M. (2000). Improved insulin-sensitivity in mice heterozygous for PPAR-gamma deficiency. J Clin Invest *105*, 287-292.

Nagy, Z. S., Lebaron, M. J., Ross, J. A., Mitra, A., Rui, H., and Kirken, R. A. (2009). STAT5 regulation of BCL10 parallels constitutive NFkappaB activation in lymphoid tumor cells. Mol Cancer *8*, 67.

Relloso, M., Puig-Kroger, A., Pello, O. M., Rodriguez-Fernandez, J. L., de la Rosa, G., Longo, N., Navarro, J., Munoz-Fernandez, M. A., Sanchez-Mateos, P., and Corbi, A. L. (2002). DC-SIGN (CD209) expression is IL-4 dependent and is negatively regulated by IFN, TGF-beta, and anti-inflammatory agents. J Immunol *168*, 2634-2643.
